# Comparative Study on Growth Performance and Rumen Digestive Metabolism Between Cull Beef Cows and Fattening Bulls

**DOI:** 10.3390/ani16193052

**Published:** 2026-09-28

**Authors:** Junjie Bao, Hao Li, Kexin Jiang, Junzhao Xu, Yonglong Wu, Wei Ji, Shuyi Li, Baoxia Qi, Huaxin Niu

**Affiliations:** College of Animal Science and Technology, Inner Mongolia Minzu University, Tongliao 028000, China; 13224847976@163.com (J.B.); 13155490537@163.com (H.L.); 13634709903@163.com (K.J.); xujunzhao1124@163.com (J.X.); wuyl09@163.com (Y.W.); xueqi1994wang@163.com (W.J.); lsy2835245597@163.com (S.L.); mdqbx@163.com (B.Q.)

**Keywords:** culled beef cows, fattening, rumen microbiome, carbohydrate-active enzymes, plasma metabolomics

## Abstract

Culled beef cows may retain practical value for fattening after reproductive use, but the rumen and host physiological characteristics associated with this response remain incompletely characterized. This study compared culled beef cows (CBC; *n* = 9) with fattening bulls (CON; *n* = 9) using 18 healthy Simmental crossbred cattle fed the same experimental total mixed ration (TMR) under identical feedlot conditions. Growth performance, body condition score (BCS), apparent nutrient digestibility, economic outcomes, blood indices, rumen fermentation, ruminal enzyme activities, rumen metagenomes, and plasma untargeted metabolomes were assessed. Compared with CON, CBC had higher final body weight, average daily gain, feed intake, and apparent digestibility of dry matter, crude protein, and neutral detergent fiber, whereas the feed-to-gain ratio did not differ significantly between groups (*p* = 0.692). BCS increased significantly from day 0 to day 60 within both cattle categories. Because different, non-cross-calibrated BCS reference frameworks were used, no between-group comparison of BCS was made. Economic analysis indicated that the value attributable to actual live-weight gain was similar between groups when within-category prices were held constant, whereas the relative economic outcome varied with feed costs and changes in live-cattle market prices. CBC also showed more propionate-oriented fermentation, greater fibrolytic enzyme activities, distinct carbohydrate-active enzyme profiles, higher insulin and leptin, and lower non-esterified fatty acids and β-hydroxybutyrate. Because lean and fat mass were not directly measured, the tissue basis of the greater weight gain remains unresolved. Overall, CBC exhibited a stronger fattening response accompanied by coordinated differences in rumen function and host metabolic profiles.

## Simple Summary

Culled beef cows are often considered to have limited production value after reproductive use, but they may still respond to the early fattening under appropriate feeding conditions. This study compared culled beef cows with fattening bulls fed the same diet under identical feedlot conditions. Culled beef cows showed greater feed intake, body-weight gain, and nutrient digestibility. Body condition increased during the fattening stage within both cattle categories. These responses were associated with stronger rumen fiber-degrading capacity, a higher ruminal propionate proportion, reduced indicators of lipid mobilization, and changes in circulating metabolites related to energy and nutrient utilization. Culled beef cows also showed higher immunoglobulin and complement concentrations without evidence of systemic inflammatory activation. However, lean and fat mass were not directly measured; therefore, the greater weight gain could not be attributed specifically to muscle or fat deposition. Overall, the results indicate that culled beef cows retain practical value for fattening, although further measurements of body composition are required to determine the tissue basis of their weight gain.

## 1. Introduction

Culled beef cows are often marketed at a relatively low value after reproductive use, but they may still have potential for fattening and body-value improvement under appropriate feeding management. Their market value is influenced by body condition, age, production background, and marketing conditions [1]. Realimentation of cull beef cows with high-concentrate diets has been shown to improve live performance, body weight recovery, and carcass-related value [2]. Feeding culled cows high-energy concentrate diets for short periods can also increase live weight, carcass weight, average daily gain (ADG), and dressing percentage, with marked responses during the early feeding period [3]. Short-term feeding programs for culled beef cows further affect live animal performance, carcass characteristics, and economic outcomes, indicating that feeding strategy is an important determinant of their fattening value [4]. Pre-finishing supplementation and feedlot adaptation influence performance and body condition in cull Nelore cows [5]. In thin cull beef cows, dietary roughage level and rumen adaptation strategy affected feedlot performance and carcass composition during a 42-day realimentation period [6], while most compensatory gain occurred during the first 28 days of feeding in another study of thin beef cull cows [7]. These findings suggest that culled beef cows may have practical short-term feeding value, especially when nutritional history and body reserve status allow a realimentation-like response.

Despite this production evidence, the biological characteristics associated with the fattening response in culled beef cows remain insufficiently understood. Previous studies have mainly evaluated live performance, carcass traits, meat quality, or economic outcomes. These endpoints are important for production decisions, but they provide limited information on the digestive and physiological characteristics associated with differences in fattening response between culled beef cows and fattening bulls fed the same diet. Culled beef cows and fattening bulls differ not only in production category but also in sex, age, reproductive history, physiological maturity, endocrine background, and body reserve status. Therefore, differences in fattening response may involve not only feed intake but also nutrient digestion, rumen carbohydrate degradation, fermentation pattern, host energy metabolism, endocrine regulation, and immune status. Direct comparisons between culled beef cows and fattening bulls under the same feeding conditions remain limited, particularly from an integrated digestive, microbial, metabolic, immune, and economic perspective.

The rumen is the key site linking dietary nutrient utilization with host productive performance. Rumen microorganisms degrade plant structural carbohydrates and convert dietary substrates into volatile fatty acids (VFAs), microbial protein, and other metabolites that contribute to host energy and nutrient supply [8]. Large-scale rumen metagenomic studies have shown that the cow rumen microbiome contains abundant microbial genomes and enzyme-coding genes involved in plant material degradation and carbohydrate utilization [9,10]. Compared with microbial taxonomic composition alone, microbial functional capacity may provide a more direct explanation for differences in feed utilization. In beef cattle, active rumen microbial functions have been linked to feed efficiency [11], and multi-omics studies in cattle further indicate that rumen microbial structure, rumen metabolites, and host metabolites jointly contribute to differences in production performance and feed efficiency [12,13]. Carbohydrate-active enzymes (CAZymes) are particularly important for understanding ruminal fiber degradation and nutrient utilization. Cellulose and hemicellulose are major structural components of plant cell walls, and their degradation in the rumen depends largely on microbial fibrolytic enzymes and related CAZyme families [14]. Metagenomic studies based on the Carbohydrate-Active enZYmes database (CAZy) have shown that dietary and microbial differences can alter rumen CAZyme profiles, reflecting changes in plant polysaccharide degradation capacity [15]. In addition, fibrolytic enzymes such as cellulase and xylanase can improve ruminal fiber digestion and increase fermentable substrate availability, thereby influencing volatile fatty acid production [16,17]. Therefore, evaluating ruminal digestive enzyme activities together with CAZy family profiles may provide functional context for differences in apparent nutrient digestibility and rumen fermentation between culled beef cows and fattening bulls.

Among ruminal fermentation products, propionate is especially important because it is a major gluconeogenic precursor in ruminants. A higher propionate proportion and a lower acetate-to-propionate ratio indicate a more glucogenic fermentation pattern. In dairy cattle, postruminal propionate infusion increased serum insulin and showed a tendency to alter hepatic expression of gluconeogenesis-related genes, including phosphoenolpyruvate carboxykinase 1 (PCK1) and glucose-6-phosphatase catalytic subunit (G6PC), supporting a feed-forward role of propionate in regulating hepatic glucose metabolism [18]. In Hanwoo steers, intraruminal propionate infusion increased serum insulin concentration and decreased serum non-esterified fatty acid (NEFA) concentration, indicating a physiological connection among ruminal propionate supply, endocrine response, and reduced lipid mobilization [19]. Therefore, ruminal fermentation differences may be closely related to systemic metabolic indicators such as insulin, leptin, NEFA, and β-hydroxybutyrate (BHBA). Host immune status may also be involved in the fattening response of culled beef cows. Nutritional and metabolic status can influence immune function because immune cells require sufficient energy substrates and nutrients for proliferation, antibody synthesis, cytokine production, and acute-phase responses [20]. Thus, changes in immunoglobulins, complement components, acute-phase proteins, and cytokines may help distinguish improved immune readiness from inflammatory activation. For culled beef cows, simultaneous evaluation of metabolic, endocrine, and immune indices may provide a more complete understanding of host physiological status during fattening.

Based on these considerations, the present study compared culled beef cows and fattening bulls fed the same diet under identical feedlot conditions. Growth performance; apparent nutrient digestibility; economic evaluation; blood biochemical, hormonal, and immunological indices; rumen fermentation parameters; and ruminal digestive enzyme activities were measured. Rumen metagenomic sequencing was used to characterize microbial community structure and CAZy functional profiles, and plasma untargeted metabolomics was used to evaluate systemic metabolic differences. This study aimed to characterize the integrated relationships among rumen microbial functions, fermentation characteristics, plasma metabolic profiles, serum indices, and host phenotypes associated with the fattening response of culled beef cows and to evaluate their practical feeding value under the present feedlot conditions.

## 2. Materials and Methods

### 2.1. Experimental Animals, Experimental Design, and Diet Composition

In total, 18 healthy Simmental crossbred cattle with similar initial body weight (416.24 ± 1.47 kg) were selected at the Agricultural and Animal Husbandry Science and Technology Demonstration Park of Inner Mongolia Minzu University. The experiment was conducted from November 2025 to January 2026. The cattle were assigned to a culled beef cow group (CBC) and a fattening bull group (CON), with nine animals in each group. The CBC group consisted of nine culled multiparous beef cows aged 3–4 years, including four second-parity cows and five third-parity cows. Their last calving occurred approximately 120–150 days before the start of the experiment, and their last weaning occurred approximately 60–90 days before the experiment. None of the cows were rebred after the last weaning. These cows had completed their reproductive use and had been removed from the breeding herd as part of routine herd replacement rather than because of disease. The CON group consisted of intact fattening bulls aged 11–12 months. All cattle were clinically healthy before the experiment, and animals with obvious disease, abnormal appetite, severe lameness, or other visible health problems were not included. This study used a comparative two-group design, with cattle category treated as the grouping factor.

The experimental period lasted 67 days, including a 7-day adaptation period and a 60-day formal feeding period, and was conducted from November 2025 to January 2026. Cattle were housed individually in a semi-open barn, and feed offered and refusals were recorded separately for each animal. According to local weather records during the experimental period, the outdoor ambient temperature ranged approximately from −26 to −15 °C. The temperature inside the barn was not continuously monitored. Both groups were exposed to the same housing and seasonal environmental conditions. Both groups were fed the same experimental total mixed ration (TMR) to provide a standardized dietary environment for comparison between the two cattle categories. The TMR had an approximate concentrate-to-forage ratio of 45:55 on an air-dry basis, a calculated net energy concentration of 6.69 MJ/kg DM, and an analyzed crude protein concentration of 11.1% DM. The ration was formulated with reference to the Chinese Beef Cattle Feeding Standard (NY/T 815-2004) [21] and the practical feeding conditions of the farm. Because the nutrient requirements of growing intact bulls and mature culled cows differ, this common experimental TMR was not intended to represent an individually optimized ration for both cattle categories. Rather, it was used to compare their production and physiological responses under the same dietary, housing, and management conditions. Cattle were fed twice daily at 06:00 and 16:00 and had free access to feed and water throughout the experiment. Average daily feed intake was calculated on a dry matter basis from the daily difference between feed offered and refusals for each animal. The ingredient composition and nutrient levels of the experimental TMR are shown in Table 1.

### 2.2. Sample Collection and Phenotypic Measurements

During the experiment, growth performance, apparent nutrient digestibility, serum biochemical indices, serum hormone indices, serum immunological indices, rumen fermentation parameters, and ruminal digestive enzyme activities were measured in all animals, with nine cattle in each group. For omics analyses, six cattle from each group were purposefully selected based on initial body weights close to the respective group mean and the absence of clinical abnormalities during the experiment. Rumen fluid samples collected from these selected cattle were used for rumen metagenomic sequencing, and plasma samples collected from the same selected cattle were used for untargeted metabolomic analysis. In CBC, the initial body-weight range was 408.45–426.43 kg for all nine animals and 412.34–420.35 kg for the six selected animals. In CON, the corresponding ranges were 405.45–426.34 kg and 410.45–422.56 kg, respectively. The mean initial body weights of the selected animals were 417.32 kg in CBC and 417.13 kg in CON. Because this omics subset was intentionally restricted to animals near the center of the within-group body-weight distribution rather than obtained by simple random sampling, the omics findings should be interpreted primarily as representing animals with body weights close to the group mean.

Body weight was recorded at the beginning and end of the formal experimental period before morning feeding. Average daily gain (ADG) was calculated from the change in body weight during the 60-day formal feeding period. Feed offered and refusals were recorded daily for each animal, and average daily feed intake (ADFI) was calculated on a dry matter basis. The feed-to-gain ratio (F/G) was calculated as the ratio of ADFI to ADG.

Body condition score (BCS) was evaluated at the beginning and end of the 60-day formal feeding period before morning feeding. A nine-point scoring system was used, with 1 representing an extremely thin condition and 9 representing an extremely fat or obese condition. For culled beef cows, BCS was assessed according to the Chinese national standard GB/T 41194–2021, Technical Specification for Body Condition Score of Beef Cows [22]. The assessment was based on visual evaluation of fat coverage and body conformation around the ribs, spine, hooks, pins, tailhead, and brisket. For fattening bulls, the same nine-point scoring scale was applied based on its previous application in young Charolais bulls [23]. All animals were evaluated by the same trained evaluator, and scores were assigned in 0.5-point increments. Because the two cattle categories were evaluated using different reference frameworks that had not been formally cross-calibrated, absolute BCS values and the magnitude of BCS change were not considered directly comparable between groups. Therefore, BCS was used only to evaluate within-group changes from day 0 to day 60.

Economic evaluation was conducted using a partial-budget approach. Total feed cost over the 60-day fattening period was calculated as average daily feed intake × TMR price × 60. The change in live-animal market value under the observed price scenario was calculated as final body weight × final live-cattle price minus initial body weight × initial live-cattle price. Partial budget was then calculated as the change in live-animal market value minus total feed cost. To separate the value of actual weight gain from the effect of changing cattle prices, an additional constant-price analysis was performed, in which the value of added live weight was calculated as body-weight gain × initial live-cattle price, and the corresponding partial budget was calculated as added-weight value minus total feed cost. A price-sensitivity analysis was further conducted using alternative initial and final live-cattle price scenarios while holding animal performance and feed cost constant.

Representative feed samples were collected on days 0, 7, and 60 using the quartering method. Five subsamples collected at each sampling time were pooled into one composite sample, dried at 65 °C for 48 h, air-equilibrated for 24 h, and then ground to pass through a 1 mm sieve for routine nutrient analysis.

The filtered rumen fluid was aliquoted into 15 mL polypropylene centrifuge tubes (LABSELECT, Beijing Labgic Technology Co., Ltd., Beijing, China), rapidly frozen in liquid nitrogen, and stored at −80 °C for subsequent analyses of rumen fermentation parameters, ruminal digestive enzyme activities, and metagenomic sequencing.

Blood samples were collected before morning feeding on day 67 via jugular venipuncture. Samples used for serum biochemical, hormonal, and immunological analyses were collected into vacuum tubes without anticoagulant, whereas samples used for plasma untargeted metabolomic analysis were collected into anticoagulant-containing vacuum tubes. Serum tubes were allowed to stand at room temperature for 30–60 min and then centrifuged at 3000–3500 r/min for 10–15 min at 4 °C. Anticoagulated blood samples were gently inverted 8–10 times immediately after collection and centrifuged at 4 °C to obtain plasma. Serum and plasma samples were aliquoted into 1.5 mL cryotubes, rapidly frozen in liquid nitrogen or on dry ice, and stored at −80 °C until analysis.

A 3-day digestion trial was conducted at the end of the formal experimental period to determine apparent nutrient digestibility using acid-insoluble ash (AIA) as an internal marker, as described previously [24]. Fecal samples were collected twice daily from each animal during the digestion trial. Approximately 1 kg of feces was collected each time and mixed thoroughly, and a 200 g subsample was taken. Each fecal subsample was mixed with 40 mL of 10% H_2_SO_4_ for nitrogen fixation, sealed, labeled, and stored at −20 °C. Feed and fecal samples were dried at 65 °C for 48 h, equilibrated for 24 h, weighed, ground, and passed through a 1 mm sieve. Dry matter (DM), crude protein (CP), crude ash, neutral detergent fiber (NDF), and acid detergent fiber (ADF) were determined. Apparent digestibility was calculated using the AIA method according to the following equation:

Apparent digestibility (%) = 100 × [1 − (c/d) × (b/a)]

where a represents the nutrient concentration in the diet (% DM), b represents the nutrient concentration in feces (% DM), c represents the AIA concentration in the diet (% DM), and d represents the AIA concentration in feces (% DM).

### 2.3. Blood Biochemical, Hormonal, and Immunological Measurements

Serum biochemical indices, including glucose (GLU), creatinine (CREA), urea, total cholesterol (TC), triglyceride (TG), non-esterified fatty acids (NEFA), β-hydroxybutyrate (BHBA), total protein (TP), albumin (ALB), aspartate aminotransferase (AST), and alanine aminotransferase (ALT), were determined using commercial assay kits (Jiangsu Aidisheng Biotechnology Co., Ltd., Yancheng, China) according to the manufacturer’s instructions. Glucose and albumin were measured by spectrophotometric methods, whereas most other biochemical indices were measured using microplate-based enzymatic or colorimetric assays. The main instruments used for biochemical measurements included an L6 ultraviolet spectrophotometer (Shanghai Youke Instrument Co., Ltd., Shanghai, China) and a SpectraMax 190 microplate reader (Molecular Devices, San Jose, CA, USA).

Serum hormone indices, including growth hormone (GH), insulin-like growth factor 1 (IGF-1), insulin (INS), leptin (LEP), estradiol (E2), and testosterone (T), were measured using commercial bovine ELISA kits (Jiangsu Meimian Industrial Co., Ltd., Yancheng, China) according to the manufacturer’s protocols. Estradiol and testosterone were determined using competitive ELISA kits, whereas the other hormone indices were determined using standard ELISA kits. Absorbance was read using an Infinite F50 microplate reader (Tecan, Shanghai, China), and plate washing was performed using a DKW-310 automatic microplate washer (Wuxi Huawei Delang Technology Co., Ltd., Wuxi, China).

Serum immunological indices, including immunoglobulin G (IgG), immunoglobulin M (IgM), immunoglobulin A (IgA), complement C3, complement C4, haptoglobin (Hp), serum amyloid A (SAA), interleukin-6 (IL-6), interleukin-10 (IL-10), and tumor necrosis factor-α (TNF-α), were determined using commercial bovine ELISA kits (Jiangsu Meimian Industrial Co., Ltd., Yancheng, China) according to the manufacturer’s instructions. Absorbance reading and plate washing were performed using the same Infinite F50 microplate reader and DKW-310 automatic microplate washer described above. All measurements were conducted under the same laboratory conditions.

### 2.4. Rumen Fermentation Parameters and Digestive Enzyme Activity Measurements

Ruminal pH was measured immediately after rumen fluid collection using a pH meter (PHSJ-3F, LEICI, Shanghai, China). For volatile fatty acid (VFA) analysis, rumen fluid samples were thawed and centrifuged at 12,000× *g* for 10 min at 4 °C. Then, 1.5 mL of the supernatant was transferred into a 2 mL centrifuge tube and mixed with 0.15 mL of 25% metaphosphoric acid. VFA concentrations were determined using a GC-8860 gas chromatograph equipped with a flame ionization detector (Beijing Beifen Tianpu Instrument Technology Co., Ltd., Beijing, China). Individual VFA concentrations were quantified by comparison with external standards (Supelco Volatile Fatty Acid Standard Mix, Sigma-Aldrich, St. Louis, MO, USA) containing acetic acid, propionic acid, isobutyric acid, butyric acid, isovaleric acid, and valeric acid. The chromatographic conditions were as follows: quartz column, 6 mm × 2 m; column temperature, 150 °C; injector temperature, 220 °C; injection volume, 1 μL; detector temperature, 280 °C; nitrogen flow rate, 30 mL/min at 200 kPa; hydrogen flow rate, 30 mL/min; and air flow rate, 300 mL/min. Total volatile fatty acids (TVFAs), acetate, propionate, butyrate, isobutyrate, valerate, and the acetate-to-propionate ratio were calculated.

### 2.5. Rumen Metagenomic Sequencing and Functional Annotation

A total of 12 rumen fluid samples, each obtained from a different animal, were used for metagenomic sequencing, including six purposefully selected animals from the CON group and six from the CBC group, as described in Section 2.2. All downstream metagenomic analyses were conducted using the individual animal as the experimental unit, with six biological replicates per group. Total microbial genomic DNA was extracted from rumen fluid samples stored at −80 °C using an MP Biomedicals DNA extraction kit (MP Biomedicals, Santa Ana, CA, USA). DNA concentration and purity were evaluated using a NanoDrop 2000 spectrophotometer (Thermo Fisher Scientific, Waltham, MA, USA), and DNA integrity was assessed by 1% agarose gel electrophoresis.

Qualified DNA samples were fragmented, end-repaired, and used to construct paired-end sequencing libraries. Library quality and concentration were evaluated using agarose gel electrophoresis and qPCR. Qualified libraries were sequenced on the Illumina NovaSeq platform by Majorbio Bio-Pharm Technology Co., Ltd. (Shanghai, China). Raw sequencing reads were quality-filtered using fastp v0.20.0 to remove low-quality sequences. Host-derived contaminating sequences were removed using BWA v0.7.17 against the bovine reference genome. High-quality clean reads were assembled using MEGAHIT v1.2.9, and contigs shorter than 500 bp were discarded. Open reading frames were predicted using Prodigal v2.6.3, and predicted genes with nucleic acid lengths of ≥100 bp were retained and translated into amino acid sequences.

A non-redundant gene catalog was constructed using CD-HIT v4.6.1 with 90% sequence identity and 90% coverage. Clean reads from each sample were mapped back to the non-redundant gene catalog using SOAPaligner v2.21 with an identity threshold of 95% to estimate gene abundance. Taxonomic annotation was performed by aligning the predicted amino acid sequences against the NR database using DIAMOND v2.0.13 with a BLASTP e-value cutoff of 1 × 10^−5^. Functional annotation was performed by aligning the predicted amino acid sequences against the KEGG database using DIAMOND v2.0.13 with the same e-value cutoff. Carbohydrate-active enzyme annotation was performed by aligning the predicted amino acid sequences against the CAZy database using HMMER v3.1b2 with an e-value cutoff of 1 × 10^−5^.

The relative abundances of microbial taxa, KEGG functional pathways, CAZy classes, and representative CAZy families were calculated for downstream comparisons between the CON and CBC groups. Beta-diversity was evaluated using principal coordinate analysis (PCoA) based on Bray–Curtis distances, and group separation was tested using analysis of similarity (ANOSIM). Differences in microbial taxa, KEGG functional pathways, CAZy classes, and representative CAZy families between groups were evaluated at the animal level using the Wilcoxon rank-sum test, with six biological replicates per group. For comparisons of microbial taxa, KEGG functional pathways, CAZy classes, and representative CAZy families, *p*-values were adjusted separately within each analysis family using the Benjamini–Hochberg procedure to control the false discovery rate (FDR). FDR-adjusted q-values were used to determine statistical significance, with q < 0.05 considered significant. Raw *p*-values are reported in the corresponding figures and tables for descriptive transparency. The rumen metagenomic sequencing data have been deposited in the NCBI Sequence Read Archive under BioProject accession number PRJNA1481135.

### 2.6. Plasma Untargeted Metabolomic Analysis

A total of 12 plasma samples, each obtained from a different animal, were used for untargeted metabolomic analysis, including six purposefully selected animals from the CON group and six from the CBC group, as described in Section 2.2. All downstream metabolomic analyses were conducted using the individual animal as the experimental unit, with six biological replicates per group.

Plasma samples were mixed with extraction solvent, treated by low-temperature ultrasonication, and centrifuged. The supernatant was collected, dried under nitrogen, reconstituted, and then subjected to LC-MS/MS analysis. Quality control (QC) samples were prepared by pooling equal volumes of all plasma samples and were used to evaluate analytical stability and repeatability.

Chromatographic separation was performed using an HSS T3 column (100 mm × 2.1 mm, 1.8 μm). The flow rate was 0.40 mL/min, the column temperature was maintained at 40 °C, and the injection volume was 3 μL. Mass spectrometric data were acquired using an electrospray ionization source in both positive and negative ion modes.

Raw LC-MS/MS data were processed using Progenesis QI v3.0 (Waters Corporation, Milford, MA, USA) for peak detection, peak extraction, retention time alignment, and peak integration. Metabolite annotation was performed by matching the detected features against the Human Metabolome Database (HMDB), Metlin database, and Majorbio in-house database. Annotation confidence was further categorized according to the Majorbio platform-specific scheme. Level B(i) indicates matching against experimental MS/MS spectral libraries, including in-house or commercial libraries, whereas level B(ii) indicates matching against computer-simulated theoretical MS/MS spectral libraries. These annotations were based on spectral-library matching and were not confirmed using authentic reference standards. The annotation level of each key differential metabolite is reported with the corresponding key-metabolite results. To reduce technical variation, the annotated data matrix was preprocessed by removing features with missing values greater than 20% within each group, imputing missing values with the minimum value across all samples, normalizing peak intensities using total-sum normalization, removing variables with a relative standard deviation greater than 30% in QC samples, and applying log10 transformation.

The processed data matrix was uploaded to the Majorbio Cloud Platform (https://cloud.majorbio.com, accessed on 22 September 2026) for subsequent statistical analysis and visualization. After quality control, peak intensities from the three LC–MS/MS injections for each animal were averaged to generate a single animal-level metabolomic profile. All subsequent statistical analyses were performed using the individual animal as the experimental unit, with six biological replicates per group (*n* = 6); technical injections were not treated as independent observations. Principal component analysis (PCA) and partial least squares discriminant analysis (PLS-DA) were used to evaluate the overall metabolic distribution between groups. The reliability of the PLS-DA model was evaluated using 200 permutation tests. Between-group differences in individual metabolites were evaluated using Student’s *t*-test, and *p*-values were adjusted for multiple testing using the Benjamini–Hochberg procedure to control the false discovery rate (FDR). Differential metabolites were defined as those with an FDR-adjusted *p*-value < 0.05 and a PLS-DA variable importance in projection (VIP) value > 1. Key differential metabolites were further selected using the stricter criteria of an FDR-adjusted *p*-value < 0.05, VIP > 1, and FC > 1.20 or FC < 0.83. KEGG pathway enrichment analysis was performed using the differential metabolites identified by FDR-adjusted *p*-value < 0.05 and VIP > 1, and pathway-level *p*-values were further adjusted using the Benjamini–Hochberg method. Pathways with an adjusted *p*-value < 0.05 were considered significantly enriched.

### 2.7. Statistical Analysis and Multi-Omics Association Analysis

Phenotypic data were preprocessed using Excel 2021 and statistically analyzed using SPSS 27.0 (IBM Corp., Armonk, NY, USA). For growth performance, apparent nutrient digestibility, blood biochemical indices, serum hormone indices, serum immunological indices, rumen fermentation parameters, and ruminal digestive enzyme activities, individual animals were used as the experimental units, with nine biological replicates per group. Before group comparisons, normality and homogeneity of variance were evaluated using the Shapiro–Wilk test and Levene’s test, respectively. When data met the assumptions of normal distribution and equal variance, independent-samples *t*-tests were used to compare differences between the CON and CBC groups. Otherwise, non-parametric tests were applied. Because BCS was recorded on an ordinal nine-point scale at 0.5-point intervals and different reference frameworks were used for the two cattle categories, no between-group statistical comparisons of BCS were performed. Within each group, changes in BCS from day 0 to day 60 were evaluated using a two-sided Wilcoxon signed-rank test, with the individual animal as the paired experimental unit. BCS data are presented descriptively as mean ± standard error of the mean for consistency with the other phenotypic variables. Differences were considered statistically significant at *p* < 0.05. For the 27 blood biochemical, hormonal, and immunological indices, multiple testing was addressed by applying the Benjamini–Hochberg procedure across all 27 between-group comparisons as a single family. Both the raw *p*-values and the corresponding false discovery rate (FDR)-adjusted q-values are reported. For these blood indices, statistical significance after correction was defined as q < 0.05. *p*-values for the other prespecified phenotypic outcomes, including growth performance, apparent nutrient digestibility, rumen fermentation parameters, and ruminal digestive enzyme activities, were not adjusted for multiple testing and are reported as raw *p*-values. For high-dimensional omics analyses, multiple-testing correction was applied separately within the corresponding analysis as described in the relevant Methods sections.

Spearman correlation analysis was used to evaluate associations among representative CAZy families, key differential plasma metabolites, rumen fermentation parameters, ruminal digestive enzyme activities, blood indices, apparent nutrient digestibility, and growth performance traits. Correlation heatmaps were used to visualize relationships among rumen microbial carbohydrate-active functions, systemic metabolic responses, and host phenotypic traits. For correlation analyses involving omics variables, only the 12 animals included in the omics subset were used (*n* = 6 per group), and omics data were matched with the corresponding phenotypic measurements at the individual-animal level. For the Spearman correlation analyses, *p*-values were adjusted within each correlation matrix using the Benjamini–Hochberg method to control the false discovery rate (FDR).

## 3. Results

### 3.1. Growth Performance, Apparent Nutrient Digestibility, Body Condition Score, and Economic Evaluation

As shown in Table 2, initial body weight did not differ between CON and CBC, indicating comparable baseline body weight between the two groups. Compared with CON, CBC had higher final body weight, average daily gain (ADG), and average daily feed intake (ADFI) (*p* < 0.01 or *p* < 0.001), whereas the feed-to-gain ratio (F/G) did not differ between groups (*p* > 0.05). CBC also had higher apparent digestibility of dry matter (DM), crude protein (CP), and neutral detergent fiber (NDF) than CON (*p* < 0.05). Apparent digestibility of acid detergent fiber (ADF) tended to be higher in CBC (*p* = 0.06). These results indicate that CBC showed greater short-term growth response and higher apparent digestibility of DM, CP, and NDF, without a significant change in F/G.

As shown in Table 3, BCS increased significantly from day 0 to day 60 within both groups. In CON, BCS increased from 5.44 ± 0.19 to 7.06 ± 0.21 (*p* = 0.004), whereas in CBC, BCS increased from 3.56 ± 0.18 to 7.22 ± 0.15 (*p* = 0.004). The descriptive changes in BCS were 1.61 ± 0.18 and 3.67 ± 0.20 points in CON and CBC, respectively. Because different, non-cross-calibrated BCS reference frameworks were used for the two cattle categories, absolute BCS values and the magnitude of BCS change were not statistically compared between groups.

As shown in Table 4, CBC had higher ADG and ADFI than CON, resulting in a higher daily feed cost. Although CBC had a higher daily gain revenue, its daily gain margin was lower than that of CON (7.26 vs. 8.36 RMB/head/day). Under the observed market-price scenario, the change in live-animal market value was greater in CBC than in CON. However, this difference reflected both live-weight gain and changes in live-cattle prices over the feeding period. When the within-category live-cattle price was held constant and only the value of actual live-weight gain was considered, the added-weight values were similar between groups (1416.00 RMB/head for CBC and 1400.00 RMB/head for CON). However, after feed costs were deducted, the constant-price partial budget was lower in CBC than in CON (471.18 vs. 606.56 RMB/head). This result was consistent with the lower daily gain margin (7.26 vs. 8.36 RMB/head/day) and 60-day gain margin (435.60 vs. 501.60 RMB/head) in CBC. The price-sensitivity analysis (Table S1) further showed that the relative economic outcomes varied across the market-price scenarios evaluated. Thus, comparable value generated from added live weight did not translate into comparable net partial-budget return.

### 3.2. Blood Indices, Rumen Fermentation, and Ruminal Enzyme Activities

Blood biochemical, hormonal, and immunological indices are shown in Table 5. After Benjamini–Hochberg FDR correction across the 27 blood indices, all between-group differences that were significant based on the raw *p*-values remained significant at q < 0.05. Among serum biochemical indices, CBC had lower non-esterified fatty acid (NEFA) and β-hydroxybutyrate (BHBA) concentrations than CON, whereas urea and total cholesterol (TC) were higher in CBC. The difference in BHBA remained significant after FDR correction (raw *p* = 0.012, q = 0.023). No significant differences were observed in glucose, creatinine, total protein, albumin, aspartate aminotransferase, alanine aminotransferase, or triglycerides between the two groups.

For serum hormone indices, CBC had higher insulin, leptin, and estradiol than CON, whereas growth hormone and testosterone were lower in CBC than in CON. The difference in estradiol remained significant after FDR correction (raw *p* = 0.014, q = 0.025). Insulin-like growth factor 1 did not differ significantly between groups.

For serum immunological indices, CBC had higher IgG, IgM, IgA, complement C3, complement C4, and IL-10 than CON after FDR correction (q < 0.05), whereas haptoglobin, serum amyloid A, IL-6, and TNF-α did not differ between groups. These results indicate that the between-group differences in serum biochemical, hormonal, and immunological profiles were robust to FDR correction.

Rumen fermentation parameters and ruminal enzyme activities are shown in Figure 1. Compared with CON, CBC had higher total volatile fatty acid concentrations and propionate proportion, but a lower acetate-to-propionate ratio (*p* < 0.05 or *p* < 0.001). CBC also had higher cellulase, xylanase, β-glucosidase, and amylase activities than CON (*p* < 0.01 or *p* < 0.001). These results indicate that CBC showed a distinct rumen fermentation profile characterized by a higher total VFA concentration and propionate proportion, together with higher carbohydrate-degrading enzyme activities.

### 3.3. Differences in Rumen Microbial Community Structure and CAZy Functional Background Between CBC and CON

As shown in Figure 2A, the rumen microbial community at the genus level was dominated by *Prevotella*, *Xylanibacter*, Candidatus *Cryptobacteroides*, *Bacteroides*, *Ruminococcus*, and taxa grouped as “others” in both CON and CBC. Compared with CON, CBC showed a relatively lower proportion of *Prevotella* and *Xylanibacter*, whereas the proportion of taxa grouped as “others” appeared higher. These results indicate that the dominant rumen microbial composition differed between the two groups at the genus level.

The PCoA analysis further showed a clear separation of rumen microbial communities between CON and CBC (R = 0.752, *p* = 0.002), indicating that the CBC group was associated with a distinct rumen microbial community structure compared with CON (Figure 2B).

At the CAZy class level, both groups were dominated by glycoside hydrolases, followed by glycosyl transferases, carbohydrate esterases, and carbohydrate-binding modules, whereas polysaccharide lyases, auxiliary activities, and cellulosome modules accounted for relatively small proportions (Figure 2C). Differential abundance analysis further showed that several CAZy classes differed significantly between CON and CBC (Figure 2D). Glycoside hydrolases, carbohydrate-binding modules, polysaccharide lyases, auxiliary activities, and cellulosome modules were higher in CBC, whereas glycosyl transferases were higher in CON. Carbohydrate esterases showed no significant difference between the two groups.

Overall, these results indicate that CBC was associated not only with structural differentiation of the rumen microbial community but also with broad remodeling of carbohydrate-active functional backgrounds. This provided the basis for the subsequent analysis of representative CAZy families.

### 3.4. Directional Remodeling of Representative CAZy Families and Their Major Contributing Genera

As shown in Figure 3A and Table 6, the six representative CAZy families showed different abundance patterns between CON and CBC. GH2 was higher in CON than in CBC (70.34% vs. 66.63%, *p* = 0.004), whereas GH78, CBM67, CBM4, and CE6 were higher in CBC than in CON (11.07% vs. 9.34%, *p* = 0.012; 4.06% vs. 3.40%, *p* = 0.016; 3.66% vs. 3.17%, *p* < 0.001; and 3.42% vs. 2.99%, *p* = 0.005, respectively). GH106 tended to be higher in CBC than in CON (11.15% vs. 10.74%, *p* = 0.060).

As shown in Figure 3B and Table 7, the six representative CAZy families were mainly contributed by several dominant genera, including *Prevotella*, *Xylanibacter*, *Bacteroides*, and taxa grouped as “Others”. *Prevotella* was the top contributor to GH2, GH106, CBM4, and CE6, with contribution values of 1795.22, 247.27, 109.10, and 77.20, respectively. *Bacteroides* ranked among the top three contributors to GH106, GH78, and CBM67, whereas *Xylanibacter* ranked among the top contributors to GH2, CBM4, and CE6. In addition, “Others” accounted for a substantial fraction of GH78 and CBM67.

### 3.5. Overall Differences in Plasma Metabolomic Profiles, Differential Metabolites, and Enriched Metabolic Pathways Between CON and CBC

As shown in Figure 4A, the plasma metabolomic profiles of CON and CBC were clearly separated in the PLS-DA model based on six biological replicates per group. The permutation test yielded a negative Q^2^ intercept (−0.449), indicating that the predictive performance of the permuted models was lower than that of the original model and providing no clear evidence of predictive overfitting (Figure 4B).

Using an FDR-adjusted *p*-value < 0.05 and VIP > 1 as the screening criteria, 252 differential plasma metabolites were identified between CON and CBC, including 114 metabolites with higher abundance and 138 with lower abundance in CBC. Under the stricter criteria of an FDR-adjusted *p*-value < 0.05, VIP > 1, and FC > 1.20 or FC < 0.83, 14 key differential plasma metabolites were identified and visualized in the volcano plot (Figure 4C). Representative key metabolites are summarized in Table 8.

KEGG pathway enrichment analysis based on the 252 differential metabolites identified five pathways that remained significant after Benjamini–Hochberg correction (adjusted *p* < 0.05), including bile secretion, histidine metabolism, serotonergic synapse, arachidonic acid metabolism, and asthma (Figure 4D). Among these pathways, bile secretion contained the largest number of mapped differential metabolites.

### 3.6. Correlation Analysis Among CAZy Families, Differential Metabolites, Blood Indices, Rumen Function, and Growth Phenotypes

To further integrate rumen microbial functional changes, plasma metabolic responses, rumen fermentation, digestive enzyme activities, blood indices, nutrient digestibility, and growth performance, Spearman correlation analysis was performed and visualized using correlation heatmaps. As shown in Figure 5A, representative CAZy families displayed distinct correlation patterns with digestive enzyme activities, rumen fermentation parameters, blood indices, nutrient digestibility, and growth traits. CBC-associated CAZy families, including GH78, GH106, CBM67, CBM4, and CE6, were generally positively correlated with TVFA concentration, β-glucosidase, cellulase, xylanase, propionate proportion, INS, IL-10, ADG, and NDF digestibility but negatively correlated with BHBA, NEFA, and the acetate-to-propionate ratio. In contrast, GH2 generally showed the opposite pattern, with negative correlations with TVFA concentration, carbohydrate-degrading enzyme activities, propionate proportion, INS, IL-10, ADG, and NDF digestibility and positive correlations with BHBA, NEFA, and the acetate-to-propionate ratio.

Key differential plasma metabolites also showed distinct associations with phenotypic traits (Figure 5B). The CBC-enriched metabolites 11β-hydroxyandrosterone-3-glucuronide, methionyl-leucylphenylalanine, and (4E,6Z)-3-hydroxydeca-4,6-dienoylcarnitine were generally positively correlated with TVFA concentration, NDF digestibility, ADG, propionate proportion, IL-10, cellulase, xylanase, β-glucosidase, and INS but negatively correlated with BHBA, NEFA, and the acetate-to-propionate ratio. In contrast, metabolites relatively higher in CON, including imidazole-4-acetaldehyde, 6-hydroxyindole sulfate, Bilirubin, and 2-methoxy-estradiol-17β 3-glucuronide, generally showed the opposite correlation pattern. These metabolites were positively associated with BHBA, NEFA, and the acetate-to-propionate ratio but negatively associated with TVFA concentration, NDF digestibility, ADG, propionate proportion, IL-10, carbohydrate-degrading enzyme activities, and INS.

Together, these correlation patterns showed that CBC-associated CAZy families and plasma metabolites were directionally aligned with ruminal carbohydrate-degrading enzyme activities, propionate proportion, NDF digestibility, endocrine and immune indices, and growth traits. Given the limited number of biological replicates used for the omics analyses, these correlation analyses should be regarded as exploratory and hypothesis-generating rather than as confirmatory evidence of biological relationships.

### 3.7. Integrated Microbiome–Metabolite–Host Phenotype Associations Related to the Fattening Response of Culled Beef Cows

Based on the integrated results of growth performance, apparent nutrient digestibility, rumen fermentation, digestive enzyme activities, rumen metagenomics, plasma metabolomics, and correlation analysis, a descriptive microbiome–CAZyme–fermentation–metabolite–host phenotype framework was constructed to summarize the coordinated differences observed between CBC and CON (Figure 6).

Under the same dietary conditions, CBC showed differences in rumen microbial structure and carbohydrate-active enzyme profiles compared with CON. The CAZy families that were relatively enriched or increased in CBC, including GH78, GH106, CBM67, CBM4, and CE6, were associated with functions related to plant cell-wall degradation, substrate binding, de-esterification, and oligosaccharide utilization. These patterns were observed together with higher activities of cellulase, xylanase, β-glucosidase, and amylase in rumen fluid. CBC also exhibited higher total volatile fatty acid concentration and propionate proportion and a lower acetate-to-propionate ratio, indicating a more propionate-oriented fermentation profile.

At the host level, CBC showed lower NEFA and BHBA concentrations together with higher insulin, leptin, IL-10, and immunoglobulin concentrations. Plasma metabolomic differences were further characterized by five KEGG pathways that remained significantly enriched after Benjamini–Hochberg correction, including bile secretion, histidine metabolism, serotonergic synapse, arachidonic acid metabolism, and asthma. Collectively, these findings describe coordinated associations among rumen microbial functions, fermentation characteristics, plasma metabolic profiles, endocrine and immune indices, and growth-related phenotypes in CBC and CON. These relationships are descriptive and associative and should not be interpreted as evidence of a causal pathway. Therefore, the observed fattening response of culled beef cows was characterized by coordinated differences in rumen microbial carbohydrate-active functions, propionate-oriented fermentation, plasma metabolic profiles, and host growth-related phenotypes.

## 4. Discussion

### 4.1. Growth, Nutrient Utilization, Body Condition, and Economic Response

In the present study, culled beef cows (CBC) had higher final body weight, average daily gain (ADG), and average daily feed intake (ADFI) than fattening bulls (CON) when both groups were fed the same experimental total mixed ration (TMR) under identical feedlot conditions.

CBC also showed higher apparent digestibility of dry matter (DM), crude protein (CP), and neutral detergent fiber (NDF), whereas the feed-to-gain ratio (F/G) did not differ between groups. These results indicate that the stronger fattening response of CBC was mainly related to greater feed intake and improved nutrient digestibility, rather than to a clear improvement in feed conversion efficiency.

BCS increased significantly from day 0 to day 60 within both cattle categories, indicating an improvement in external body condition during the fattening period within each group. However, the BCS assessments for culled beef cows and fattening bulls were based on different reference frameworks. Although both frameworks used a nine-point numerical scale and all animals were evaluated by the same trained evaluator, the two scoring systems were not formally cross-calibrated. Therefore, absolute BCS values and the magnitude of BCS change cannot be interpreted as directly comparable between CON and CBC. Accordingly, BCS was used only to characterize within-group changes in body condition and not to infer a greater body-condition response in either cattle category. However, BCS is a semiquantitative indicator of body condition and cannot directly partition body weight gain into adipose and lean tissues. A recent study of cull beef cows showed that cows with an average BCS of 4–6 had greater backfat thickness and ribeye area than cows with a thin BCS of 1–3 [25]. Earlier carcass-based research also showed that, as BCS increased from 2 to 8, fat thickness and longissimus muscle area increased, whereas the actual muscle-to-fat ratio and predicted carcass cutability decreased [26]. Thus, a higher BCS may reflect changes in both fatness and muscling, but it cannot be used as a direct measure of lean body mass. Recent research on compensatory growth in beef cattle further demonstrated that growth and feed efficiency responses during realimentation may not correspond directly to persistent differences in body composition, particularly when animals are compared at different ages or body weights [27]. Because ultrasound backfat thickness, longissimus muscle area, lean body mass, and carcass composition were not measured in the present study, the relative contributions of adipose tissue deposition, skeletal muscle accretion, body water, and gastrointestinal fill to the observed body weight gain remain unresolved.

These findings are consistent with previous studies showing that culled beef cows can still respond to targeted short-term feeding when nutritional supply, body condition, and feedlot management are appropriate. Realimentation of cull beef cows with high-concentrate diets has been reported to improve live performance, body weight recovery, and carcass-related traits [2]. Feeding cull cows high-energy concentrate diets for short periods can also increase live weight, carcass weight, ADG, and dressing percentage, with marked responses during the early feeding period [3]. Short-term feeding programs for culled beef cows further affect live animal performance, carcass characteristics, and economic outcomes [4]. In addition, pre-finishing supplementation and feedlot adaptation can influence feedlot performance and body condition in cull cows [5], while studies in thin cull beef cows showed that dietary roughage level, rumen adaptation strategy, and days on feed can affect feedlot performance and compensatory gain [6,7]. These previous findings support the present observation that culled beef cows should not be regarded only as terminal animals with limited feeding value; rather, they can show a measurable fattening response under an appropriate nutritional program.

The higher apparent digestibility of DM, CP, and NDF in CBC was biologically consistent with their greater growth response. Although CBC consumed more feed and gained more weight, the feed-to-gain ratio did not differ significantly between groups (11.1 vs. 10.9; *p* = 0.692). Thus, the greater weight gain of CBC was achieved primarily through greater feed intake rather than through a demonstrable improvement in feed conversion efficiency. The observed differences should, however, be interpreted within the nutritional and environmental context of the present experiment. The CBC entered the experiment approximately 120–150 days after their last calving and 60–90 days after weaning, and none had been rebred after weaning. Therefore, their growth and metabolic responses should be interpreted as those of nonpregnant, post-weaning culled cows at this specific reproductive stage and should not be generalized to cows culled immediately after weaning or at other stages of the reproductive cycle. The common experimental TMR was provided to both groups to standardize dietary exposure and was not individually optimized for the distinct nutrient requirements of growing intact bulls and mature culled cows. In addition, the experiment was conducted during the cold season in Inner Mongolia, when outdoor temperatures were approximately −26 to −15 °C, and cattle were housed in a semi-open barn. Such cold conditions may have increased maintenance energy requirements. Because CBC and CON differed in cattle category, age, sex, physiological status, reproductive history, endocrine background, and body reserve characteristics, the magnitude of the additional energy demand imposed by cold exposure, as well as the response to the common diet, may not have been identical between the two groups. Therefore, the observed differences in feed intake, nutrient digestibility, and growth performance should be interpreted as production-category-associated responses under the specific common-diet and winter environmental conditions of this study, rather than as evidence that one cattle category had intrinsically superior performance under an individually optimized feeding program.

The economic evaluation indicates that the practical value of fattening for CBC depends strongly on the accounting approach and changes in live-cattle market prices. Under the observed market-price scenario, CBC showed a larger increase in live-animal market value and a more favorable whole-period partial budget than CON. However, the observed difference in market-value change reflected both actual live-weight gain and changes in unit cattle prices between the beginning and end of the feeding period. When live-cattle prices were held constant within each cattle category, the value generated from actual live-weight gain was similar between CBC and CON. However, after feed costs were deducted, the constant-price partial budget was lower in CBC than in CON (471.18 vs. 606.56 RMB/head). This result was consistent with the lower daily gain margin and 60-day gain margin in CBC. Therefore, comparable value generated from added live weight should not be interpreted as comparable net economic return. Cull-cow market value can vary with body and carcass traits, farm type, breed, age, and calving-to-culling interval [1]. Therefore, the economic outcome observed in the present study should be interpreted within the local market-price setting and the 60-day fattening stages. The price-sensitivity analysis further showed that the relative economic ranking of the two cattle categories changed across alternative price scenarios. Thus, the apparent economic advantage of CBC under the observed market conditions should not be attributed solely to superior animal performance. Rather, CBC generated a comparable value from live-weight gain, and its overall economic outcome could be favorable under certain market-price conditions.

Overall, CBC exhibited a stronger fattening response than CON under the same feeding conditions, as reflected by higher ADG, ADFI, and apparent nutrient digestibility. The economic analysis indicated that CBC generated a comparable value from actual live-weight gain, although the relative economic outcome varied with feed costs and live-cattle market-price conditions. These production-level findings provide the phenotypic basis for further discussing the rumen functional, metabolic, and host physiological responses associated with the fattening performance of culled beef cows.

### 4.2. Rumen Fermentation and CAZyme Functional Remodeling

The improved nutrient utilization of CBC was accompanied by clear differences in rumen fermentation, ruminal digestive enzyme activities, and microbial community structure. CBC showed higher total volatile fatty acid concentration and propionate proportion, together with a lower acetate-to-propionate ratio. They also had higher cellulase, xylanase, β-glucosidase, and amylase activities. These results indicate that CBC had a rumen environment characterized by stronger carbohydrate-degrading activity and a more propionate-oriented fermentation pattern. Because rumen microorganisms degrade plant structural carbohydrates and convert dietary substrates into volatile fatty acids, microbial protein, and other metabolites that contribute to host energy and nutrient supply [8], the rumen functional differences observed in CBC were biologically consistent with their higher nutrient digestibility and growth response. Consistently, CBC and CON shared several dominant carbohydrate-utilizing genera, including *Prevotella*, *Xylanibacter*, *Bacteroides*, and *Ruminococcus*, but PCoA analysis showed clear separation between the two groups, indicating that the rumen microbial ecosystem of CBC was structurally distinct under the same dietary conditions. Large-scale rumen metagenomic studies have shown that the cow rumen microbiome contains abundant microbial genomes and enzyme-coding genes involved in plant material degradation and carbohydrate utilization [9,10]. Moreover, active rumen microbial functions have been linked to feed efficiency in beef cattle [11], and multi-omics studies indicate that rumen microbial structure, rumen metabolites, and host metabolites jointly contribute to differences in animal performance [12,13]. Therefore, the microbial difference observed here is important not only at the taxonomic level but also because it was accompanied by functional remodeling of carbohydrate-active enzymes.

At the CAZy class level, CBC showed higher glycoside hydrolases, carbohydrate-binding modules, polysaccharide lyases, auxiliary activities, and cellulosome-associated modules, whereas glycosyl transferases were higher in CON. CAZy annotation provides a functional framework for understanding enzymes and binding modules involved in glycan degradation, modification, and recognition [28]. Glycoside hydrolases cleave glycosidic bonds, polysaccharide lyases act on specific non-cellulosic polysaccharides, carbohydrate-binding modules improve enzyme access to complex substrates, and carbohydrate esterases remove acetyl or other ester substitutions that can restrict glycoside hydrolase activity [29,30]. Therefore, the CAZy class pattern in CBC suggests a coordinated shift toward substrate attachment, polysaccharide modification, backbone cleavage, and plant cell-wall deconstruction. This interpretation is consistent with the higher cellulase, xylanase, and β-glucosidase activities in CBC because cellulose and hemicellulose degradation in the rumen depends largely on microbial fibrolytic enzymes and related carbohydrate-active enzyme systems [14]. Fibrolytic enzymes can improve ruminal fiber digestion and increase fermentable substrate availability [16], while endoglucanase or xylanase supplementation has been shown to increase volatile fatty acid production from ruminant feed in ruminal in vitro cultures [17].

The representative CAZy families further clarify which carbohydrate-degrading functions were strengthened. GH78, CBM67, CBM4, and CE6 were significantly higher in CBC, and GH106 showed an increasing trend, whereas GH2 was higher in CON. GH78 and GH106 are mainly associated with microbial α-L-rhamnosidase activity, which hydrolyzes α-L-rhamnosidic linkages and removes terminal L-rhamnose residues from rhamnose-containing substrates [31]. CBM67 has been identified as an L-rhamnose-recognition module within a GH78 α-L-rhamnosidase structure [32]. Therefore, the simultaneous increase or enrichment of GH78, GH106, and CBM67 suggests enhanced recognition and hydrolysis of rhamnose-containing plant glycans in CBC. In addition, CBM4 may strengthen enzyme attachment to plant cell-wall fragments because carbohydrate-binding modules improve contact between catalytic enzymes and complex polysaccharide substrates [29]. CE6 is associated with acetyl xylan esterase activity and can contribute to the deacetylation of hemicellulosic substrates [30,33]. Thus, the higher CE6 abundance in CBC may reduce substrate shielding and facilitate the action of cellulases, xylanases, and β-glucosidases on plant cell-wall fragments.

The genus-level contribution analysis helps identify which microbial groups mainly carried the CBC-enriched CAZy functions. *Prevotella*, *Xylanibacter*, *Bacteroides*, and taxa grouped as “Others” were the major contributors to GH78, GH106, CBM67, CBM4, and CE6. Rumen and hindgut *Prevotella* species possess extensive polysaccharide utilization loci and CAZyme repertoires for degrading diverse plant polysaccharides [34]. *Xylanibacter ruminicola* can also utilize hemicelluloses, pectin, and plant storage polysaccharides through substrate-specific polysaccharide utilization loci involved in glycan sensing, binding, transport, and depolymerization [35]. The Hungate1000 project demonstrated that cultivated rumen microbial genomes encode diverse polysaccharide degradation functions, providing a genomic basis for rumen carbohydrate utilization [36]. Therefore, the contribution of *Prevotella*, *Xylanibacter*, *Bacteroides*, and less-classified taxa to CBC-enriched CAZy families suggests that the CAZy remodeling in CBC was mainly supported by plant-polysaccharide-utilizing rumen bacteria. By contrast, the higher GH2 abundance in CON should not be interpreted as evidence of stronger overall plant cell-wall degradation. GH2 is a broad glycoside hydrolase family that includes enzymes with different activities, and bovine rumen gene catalog data indicate that abundant GH families such as GH2 can act on a wide range of substrates, not necessarily from plant origin [28,37]. GH2 also includes exo-glycosidases that act on oligosaccharides produced after polysaccharide depolymerization [37]. Therefore, the higher GH2 abundance in CON may reflect terminal oligosaccharide or glycan turnover rather than stronger primary plant cell-wall deconstruction.

Overall, the rumen metagenomic results suggest that CBC had a more carbohydrate-degradation-oriented rumen functional profile than CON. This profile was characterized by microbial structural differentiation; enrichment of degradation-related CAZy classes; and increases in CAZy families involved in rhamnose-containing glycan hydrolysis, substrate binding, and xylan deacetylation. These findings provide a rumen microbial functional basis for the enhanced carbohydrate-degrading enzyme activities, propionate-oriented fermentation, and improved fiber utilization observed in culled beef cows.

### 4.3. Plasma Metabolic and Endocrine–Immune Responses

The rumen fermentation differences were accompanied by clear changes in host metabolic and endocrine indices. CBC had lower serum non-esterified fatty acid (NEFA) and β-hydroxybutyrate (BHBA) concentrations than CON, together with higher insulin and leptin concentrations. NEFA is commonly used as an indicator of energy status and lipid mobilization in beef cows [38], whereas BHBA reflects ketone body production associated with hepatic fatty acid metabolism. In ruminants, the liver takes up circulating NEFA and processes fatty acids through oxidation and ketogenesis [39]. Therefore, the lower NEFA and BHBA concentrations in CBC suggest reduced lipid mobilization pressure and lower ketogenic demand during the feeding period. The higher insulin concentration in CBC should be interpreted as part of an integrated endocrine–metabolic response rather than as an isolated cause of growth. Propionate is a major glucogenic precursor in ruminants, and previous studies showed that propionate supply can increase insulin response and decrease circulating NEFA in cattle [18,19]. In the present study, CBC showed higher propionate proportion, higher insulin, lower NEFA, and lower BHBA, while blood glucose remained unchanged. This pattern suggests stronger propionate-linked endocrine signaling and lower reliance on mobilized fatty acids, rather than a simple increase in circulating glucose. Leptin was also higher in CBC, and plasma leptin in cattle is closely related to adiposity, feeding level, energy balance, dry matter intake, growth traits, and body composition [40,41]. Thus, the higher leptin concentration in CBC may reflect differences in energy reserve status and adipose tissue signaling during fattening, but it should not be interpreted as a direct driver of feed intake or growth.

Plasma metabolomic analysis further showed distinct systemic metabolic profiles between CBC and CON. After controlling the false discovery rate at the metabolite level, KEGG enrichment analysis identified five pathways that remained significant after Benjamini–Hochberg correction, including bile secretion, histidine metabolism, serotonergic synapse, arachidonic acid metabolism, and asthma. Among these pathways, bile secretion, histidine metabolism, and arachidonic acid metabolism were considered the most directly interpretable in relation to the metabolic differences observed between the two cattle categories. Bile secretion is closely linked to hepatobiliary processing and lipid-related metabolism, and histidine metabolism reflects differences in amino acid turnover [42], whereas arachidonic acid metabolism is involved in lipid mediator-related processes [43]. In contrast, the serotonergic synapse and asthma pathways should be interpreted cautiously because KEGG pathway names may reflect shared mapped metabolites rather than the presence of neurological or respiratory disease. At the individual-metabolite level, the higher abundance of (4E,6Z)-3-hydroxydeca-4,6-dienoylcarnitine in CBC was consistent with altered fatty-acid and acyl-group handling because acylcarnitines are involved in mitochondrial fatty-acid transport and β-oxidation-related metabolism [44,45]. However, because CBC also showed lower circulating NEFA and BHBA, this metabolite should not be interpreted as evidence of greater adipose mobilization. Methionyl-leucylphenylalanine may reflect differences in peptide- and amino acid-related metabolism, whereas imidazole-4-acetaldehyde is related to histidine metabolism and has also been reported in rumen metabolomic studies of beef steers differing in feed efficiency [46]. 6-Hydroxyindole sulfate was consistent with variation in indole-related metabolism [47], whereas the steroid/glucuronide-related metabolites indicated differences in conjugative metabolic profiles. Because CBC and CON differed in sex, age, and reproductive history, the steroid/glucuronide signals should be interpreted conservatively as production-category-associated metabolic signatures rather than as evidence of nutrition-mediated endocrine mechanisms.

Taken together, the plasma metabolomic findings indicate systemic metabolic remodeling in CBC involving hepatobiliary processing, histidine-related amino acid metabolism, arachidonic acid-related lipid mediator pathways, and selected indole- and steroid/glucuronide-associated metabolites. Because AST and ALT did not differ between groups, the bile- and glucuronide-related changes are more consistent with differences in metabolic handling than with overt hepatocellular injury. These metabolic features should therefore be interpreted as associative signatures of the different physiological states of CBC and CON rather than as evidence of specific causal mechanisms.

CBC also showed higher IgG, IgM, IgA, complement C3, complement C4, and IL-10, whereas haptoglobin, serum amyloid A, IL-6, and TNF-α did not differ between groups. Nutritional and metabolic status can influence immune function because immune cells require sufficient energy substrates and nutrients for proliferation, antibody synthesis, cytokine production, and acute-phase responses [20]. Immunoglobulins are major components of humoral immunity and participate in pathogen neutralization, opsonization, complement activation, and mucosal defense [48], while complement components participate in immune surveillance, opsonization, clearance, and coordination between innate and adaptive immunity [49]. Therefore, the higher immunoglobulin and complement concentrations in CBC suggest enhanced humoral and complement-related immune readiness. However, this pattern should not be interpreted as systemic inflammation because haptoglobin and serum amyloid A are major acute-phase proteins in cattle [50], and both remained unchanged, as did IL-6 and TNF-α. The higher IL-10 concentration further suggests stronger anti-inflammatory regulation because IL-10 helps limit excessive pro-inflammatory responses and maintain immune homeostasis [51]. Taken together, CBC showed a host physiological profile characterized by lower lipid mobilization pressure, stronger propionate-linked endocrine signaling, systemic metabolic remodeling, and enhanced immune-related indices without obvious acute inflammatory activation.

### 4.4. Microbiome–Metabolite–Phenotype Associations Related to the Fattening Response of CBC

Rumen CAZyme profiles provide a functional layer between microbial community structure and host nutrient utilization because they describe the enzymatic potential for plant cell-wall deconstruction, substrate binding, polysaccharide modification, and downstream fermentation. In the present study, the integrated correlation network linked CBC-enriched CAZy families, including GH78, GH106, CBM67, CBM4, and CE6, with higher cellulase, xylanase, and β-glucosidase activities; greater NDF digestibility; higher propionate proportion; higher insulin and IL-10; and greater ADG. In contrast, these CAZy families were negatively associated with NEFA, BHBA, and the acetate-to-propionate ratio, whereas GH2 showed the opposite pattern. This coordinated pattern suggests that the main difference between CBC and CON was not a single microbial taxon or enzyme family, but a rumen functional shift linked to fiber degradation and host metabolic response. This interpretation is supported by evidence that the cow rumen contains abundant biomass-degrading genes and genomes [52], that fiber-adherent bovine rumen microbial communities are enriched in forage-specific glycoside hydrolases [53], and that genes encoding glycoside hydrolases and carbohydrate-binding modules are actively expressed during plant cell-wall polysaccharide degradation [54]. Functionally, GH78, GH106, and CBM67 have been associated with the recognition and hydrolysis of rhamnose-containing plant glycans [31,32]. CBM4 and CE6 may further support substrate binding and hemicellulose accessibility [55,56]. Therefore, their positive associations with fibrolytic enzyme activities and NDF digestibility are biologically coherent and are consistent with the enhanced fiber utilization observed in CBC.

Another component of this associative framework involves relationships among rumen carbohydrate degradation, fermentation characteristics, and host energy metabolism. In the rumen, plant carbon degradation depends on interacting microbial guilds rather than on a single organism or enzyme family, and genome-resolved multi-omics evidence shows that interspecies cross-feeding can organize carbon degradation in the rumen ecosystem [57]. In the present study, CBC-associated CAZy families were positively associated with propionate proportion and negatively associated with the acetate-to-propionate ratio, suggesting that stronger carbohydrate-active function was linked to a more propionate-oriented fermentation pattern. Volatile fatty acids are major contributors to ruminant energy supply, and propionate is the principal rumen-derived precursor for gluconeogenesis [58]. Previous cattle studies further showed that increased propionate supply can stimulate insulin response and reduce circulating NEFA without necessarily increasing basal glucose concentration [59]. This fits the present phenotype: CBC had higher propionate proportion and insulin, lower NEFA and BHBA, and unchanged glucose. Thus, the observed fermentation pattern was accompanied by a host metabolic profile characterized by stronger propionate-related endocrine signaling and lower reliance on mobilized fatty acids. The plasma metabolite correlations further support this interpretation. CBC-enriched 11β-hydroxyandrosterone-3-glucuronide, methionyl-leucylphenylalanine, and (4E,6Z)-3-hydroxydeca-4,6-dienoylcarnitine were generally positively associated with ruminal carbohydrate-degrading enzyme activities, NDF digestibility, propionate proportion, insulin, IL-10, and ADG but negatively associated with NEFA, BHBA, and the acetate-to-propionate ratio. In contrast, imidazole-4-acetaldehyde, 6-hydroxyindole sulfate, bilirubin, and 2-methoxy-estradiol-17β 3-glucuronide, which were relatively higher in CON, generally showed the opposite pattern. These associations suggest that the selected plasma metabolites reflected coordinated variation in rumen fermentation, nutrient utilization, and host metabolic status rather than isolated metabolic effects. This interpretation is consistent with multi-omics studies showing associations among rumen microbial functions, rumen metabolites, host metabolites, and performance-related phenotypes in cattle [12,13], as well as beef cattle metabolomic studies linking rumen or circulating metabolites with feed-efficiency-related traits [46,60,61].

The immune-related associations suggest that the CBC response reflected a nutrient-supported physiological state rather than inflammatory activation. CBC had higher immunoglobulins, complement components, and IL-10, whereas haptoglobin, serum amyloid A, IL-6, and TNF-α were not increased. Nutritional and metabolic status can influence immune function because immune cells depend on energy and nutrient availability for proliferation, antibody synthesis, cytokine production, and acute-phase responses [20]. Complement participates in immune surveillance and coordination of innate and adaptive immunity [49], and IL-10 is a key regulatory cytokine that limits excessive inflammatory responses [51]. Therefore, the immune profile of CBC is more consistent with enhanced immune readiness and anti-inflammatory regulation than with systemic inflammation. Overall, these results describe a coordinated microbiome–CAZyme–fermentation–metabolite–host phenotype framework associated with the fattening response of CBC. In this framework, rumen CAZy profiles were associated with carbohydrate-degrading enzyme activities and fiber utilization; these rumen functional characteristics were associated with propionate-oriented fermentation; the fermentation profile was associated with insulin and NEFA/BHBA concentrations; and systemic metabolite and immune profiles were aligned with ADG and nutrient digestibility. However, these findings should be interpreted with several important limitations. First, the proposed framework is associative and does not demonstrate that specific CAZy families, plasma metabolites, endocrine indices, or immune indices directly caused the observed differences in growth performance. Second, CBC and CON differed simultaneously in cattle category, sex, age, reproductive history, and endocrine background. Because these factors were completely confounded with group membership in the present design, their individual effects could not be statistically separated from the overall production-category effect. Accordingly, differences in endocrine indices, including testosterone and estradiol, should be interpreted primarily as characteristics of the contrasting production categories rather than as evidence of rumen- or nutrition-mediated mechanisms. Third, rumen metagenomic and plasma metabolomic analyses were conducted on six of the nine animals in each group that were purposefully selected because their initial body weights were close to the respective group mean and they remained clinically normal during the experiment. This selection narrowed the initial body-weight range represented in the omics subset and may have reduced within-group biological variability. Therefore, the rumen metagenomic and plasma metabolomic findings should be interpreted primarily as representing animals near the central portion of the body-weight distribution within each cattle category. Validation in larger cohorts using random or fully inclusive sampling will be required to confirm the robustness and generalizability of these omics patterns.

## 5. Conclusions

Under the same dietary and feedlot conditions, culled beef cows exhibited a distinct short-term fattening phenotype compared with fattening bulls, characterized by greater feed intake, weight gain, and apparent nutrient digestibility, but without a significant improvement in feed conversion efficiency. These production responses were accompanied by a coordinated rumen functional profile, including higher carbohydrate-degrading enzyme activities, greater total VFA concentration and propionate proportion, a lower acetate-to-propionate ratio, and distinct CAZy profiles. At the systemic level, lower circulating NEFA and BHBA together with higher insulin, leptin, and selected immune-related indices, as well as distinct plasma metabolic signatures, were consistent with a physiological state favoring nutrient utilization and body-weight recovery rather than extensive lipid mobilization. The integrated microbiome–metabolite–phenotype analysis therefore supports the view that the greater fattening response of CBC was associated with coordinated differences in rumen carbohydrate utilization, fermentation, and host metabolic status rather than with a single microbial or metabolic factor. From a production perspective, these findings indicate that culled beef cows can retain substantial short-term fattening potential when managed under appropriate nutritional and feedlot conditions. However, the economic value of this response was dependent on feed cost and live-cattle price conditions, and the biological basis of the additional body-weight gain could not be resolved because body composition was not directly measured. Moreover, cattle category was completely confounded with sex, age, reproductive history, physiological maturity, and endocrine background. Accordingly, the observed differences should be interpreted as production-category-associated responses under the conditions of this experiment, rather than as effects attributable specifically to culling status. Further studies using larger, more balanced designs and direct measurements of body composition are needed to determine the biological basis and broader production relevance of these responses.

## Figures and Tables

**Figure 1:**
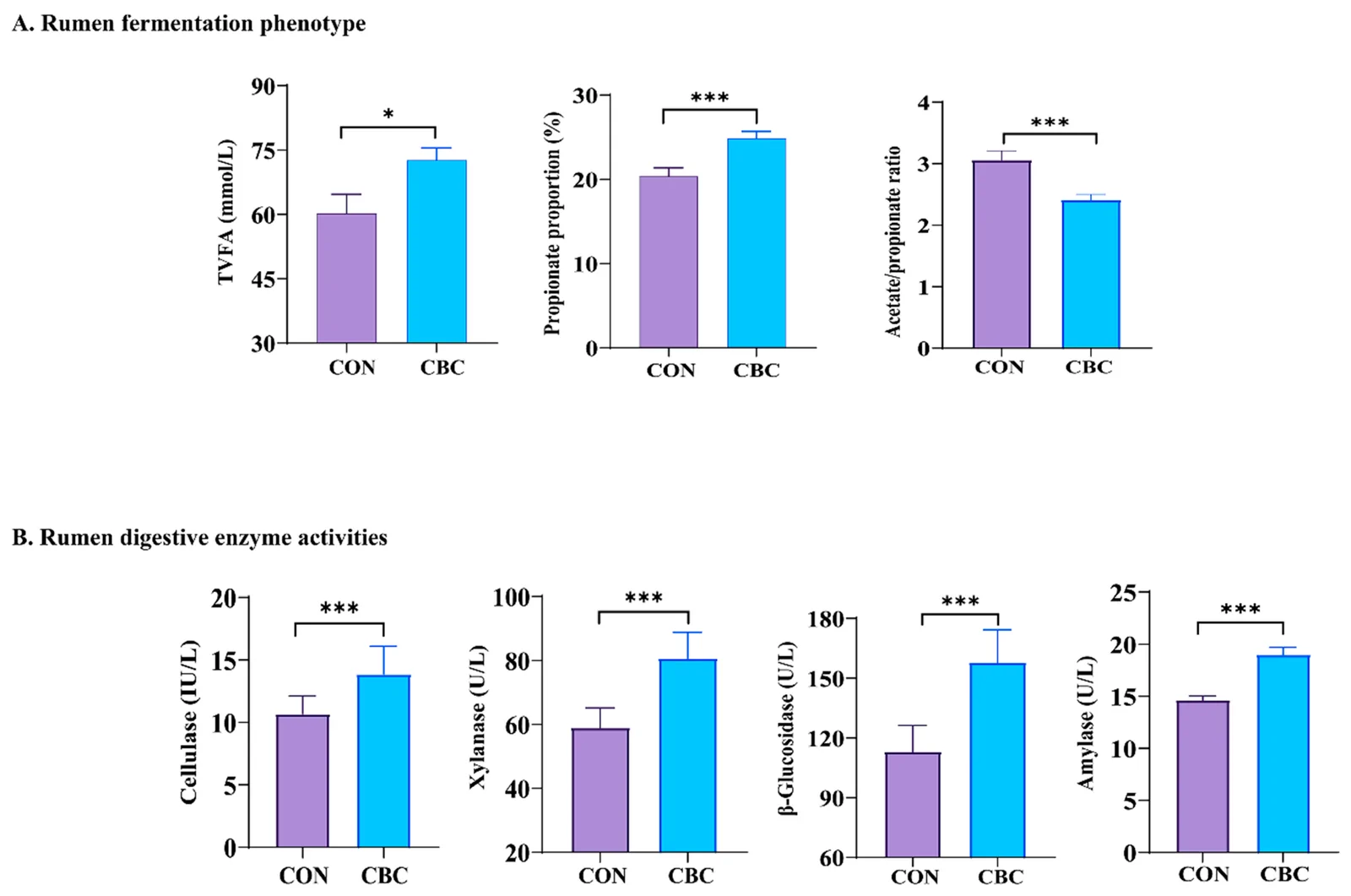
Rumen fermentation phenotype and ruminal digestive enzyme activities in CON and CBC. (**A**) Total volatile fatty acid concentration, propionate proportion, and acetate-to-propionate ratio. (**B**) Activities of cellulase, xylanase, β-glucosidase, and amylase. CON, fattening bulls; CBC, culled beef cows. Error bars represent SE. * *p* < 0.05; *** *p* < 0.001.

**Figure 2:**
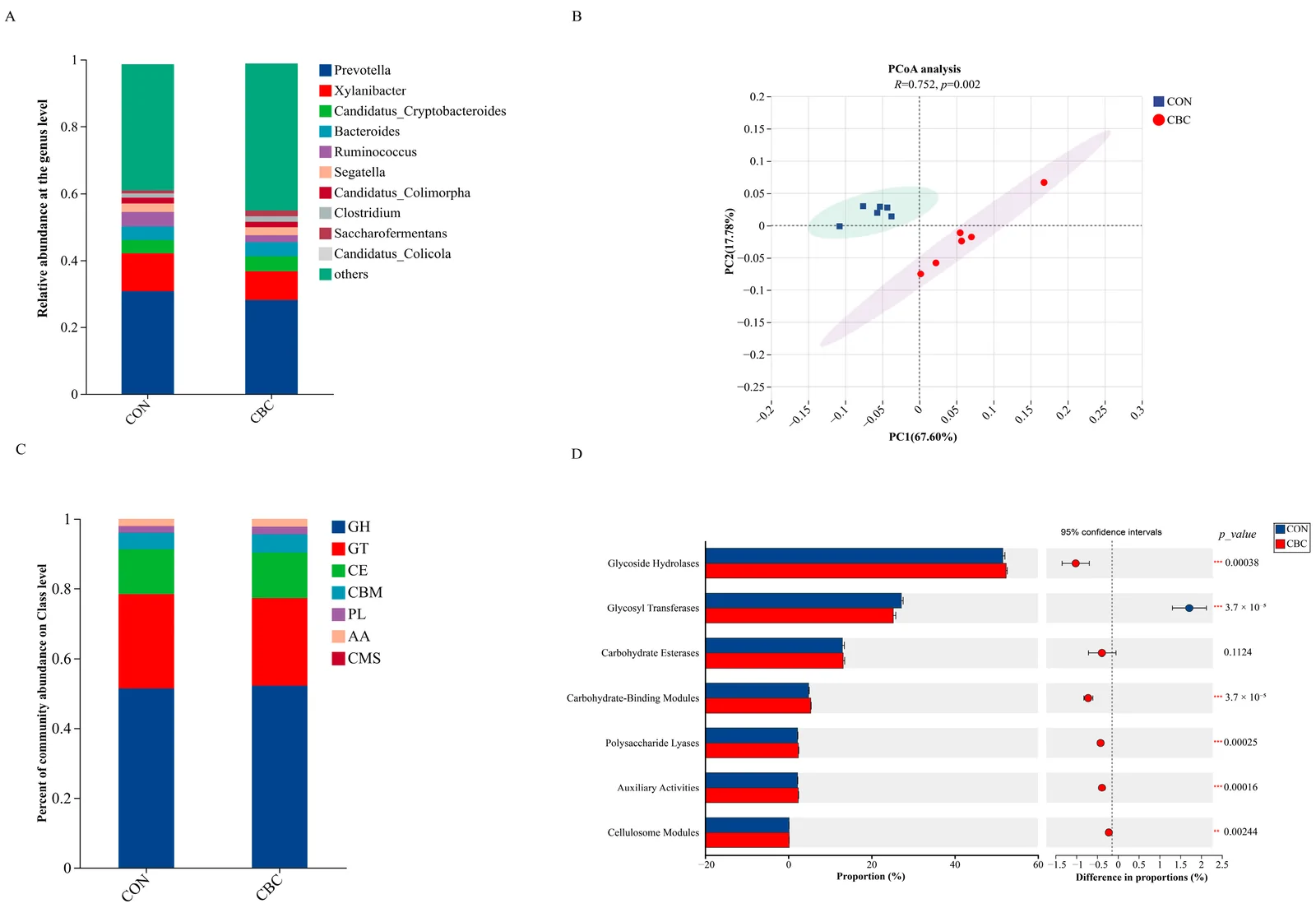
Rumen microbial structural differentiation and CAZy functional background between CON and CBC. (**A**) Relative abundance of dominant genera between CON and CBC. (**B**) PCoA analysis of rumen microbial communities based on Bray–Curtis distances. (**C**) Relative abundance of CAZy classes in the rumen metagenome. (**D**) Differential abundance of CAZy classes between CON and CBC based on the Wilcoxon rank-sum test. GH, glycoside hydrolases; GT, glycosyl transferases; CE, carbohydrate esterases; CBM, carbohydrate-binding modules; PL, polysaccharide lyases; AA, auxiliary activities; CMS, cellulosome modules. Error bars indicate 95% confidence intervals, and *p*-values are shown on the right. Red asterisks indicate significance levels (** *p* < 0.01 and *** *p* < 0.001).

**Figure 3:**
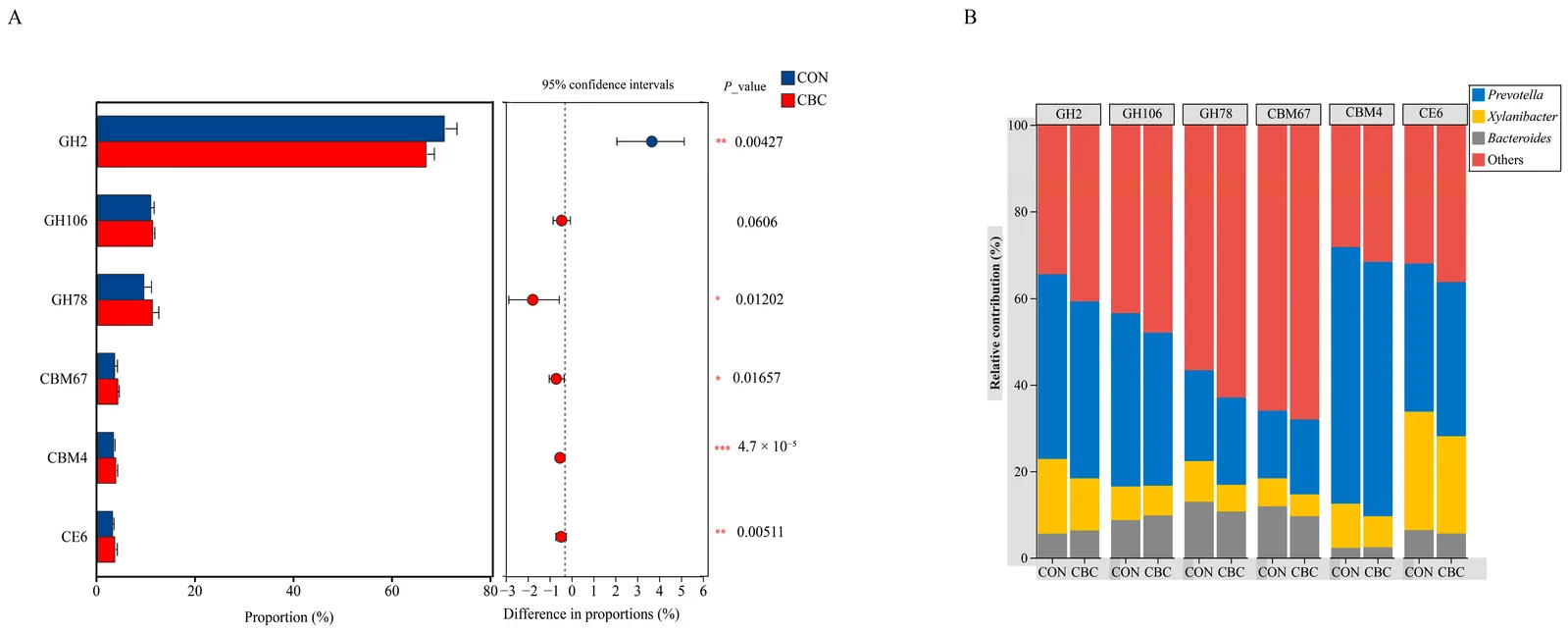
Directional remodeling and major contributing genera of representative CAZy families between CON and CBC. (**A**) Differential abundance of six representative CAZy families between CON and CBC, including GH2, GH106, GH78, CBM67, CBM4, and CE6. (**B**) Relative contribution of major genera to the six representative CAZy families in CON and CBC. Error bars in panel A indicate 95% confidence intervals, and *p*-values are shown on the right. Asterisks indicate significance levels (* *p* < 0.05, ** *p* < 0.01, and *** *p* < 0.001).

**Figure 4:**
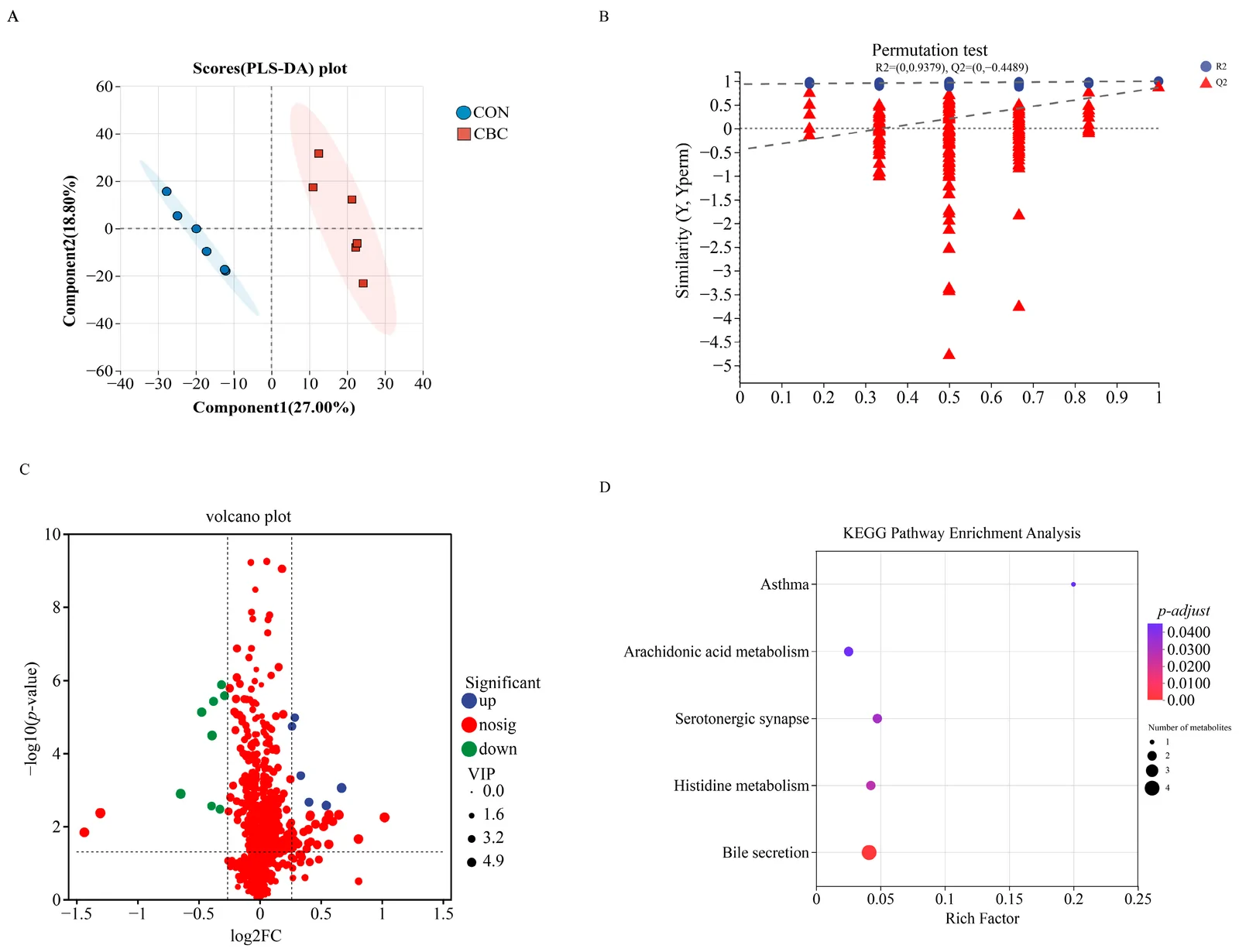
Plasma metabolomic profiles and differential metabolic pathways between CON and CBC. (**A**) PLS-DA score plot of plasma metabolomic profiles based on animal-level data (*n* = 6 biological replicates per group). (**B**) Permutation test of the PLS-DA model. (**C**) Volcano plot of key differential plasma metabolites between CON and CBC. Key differential metabolites were defined as those with an FDR-adjusted *p*-value < 0.05, VIP > 1, and FC > 1.20 or FC < 0.83. Compared with CON, metabolites with positive log2FC values were higher in CBC, whereas those with negative log2FC values were lower in CBC. (**D**) KEGG pathway enrichment analysis of differential plasma metabolites. Differential metabolites used for KEGG enrichment were screened using an FDR-adjusted *p*-value < 0.05 and VIP > 1. Only pathways with a Benjamini–Hochberg-adjusted *p*-value < 0.05 are shown. Bubble size represents the number of metabolites mapped to each pathway, and bubble color represents the adjusted *p*-value.

**Figure 5:**
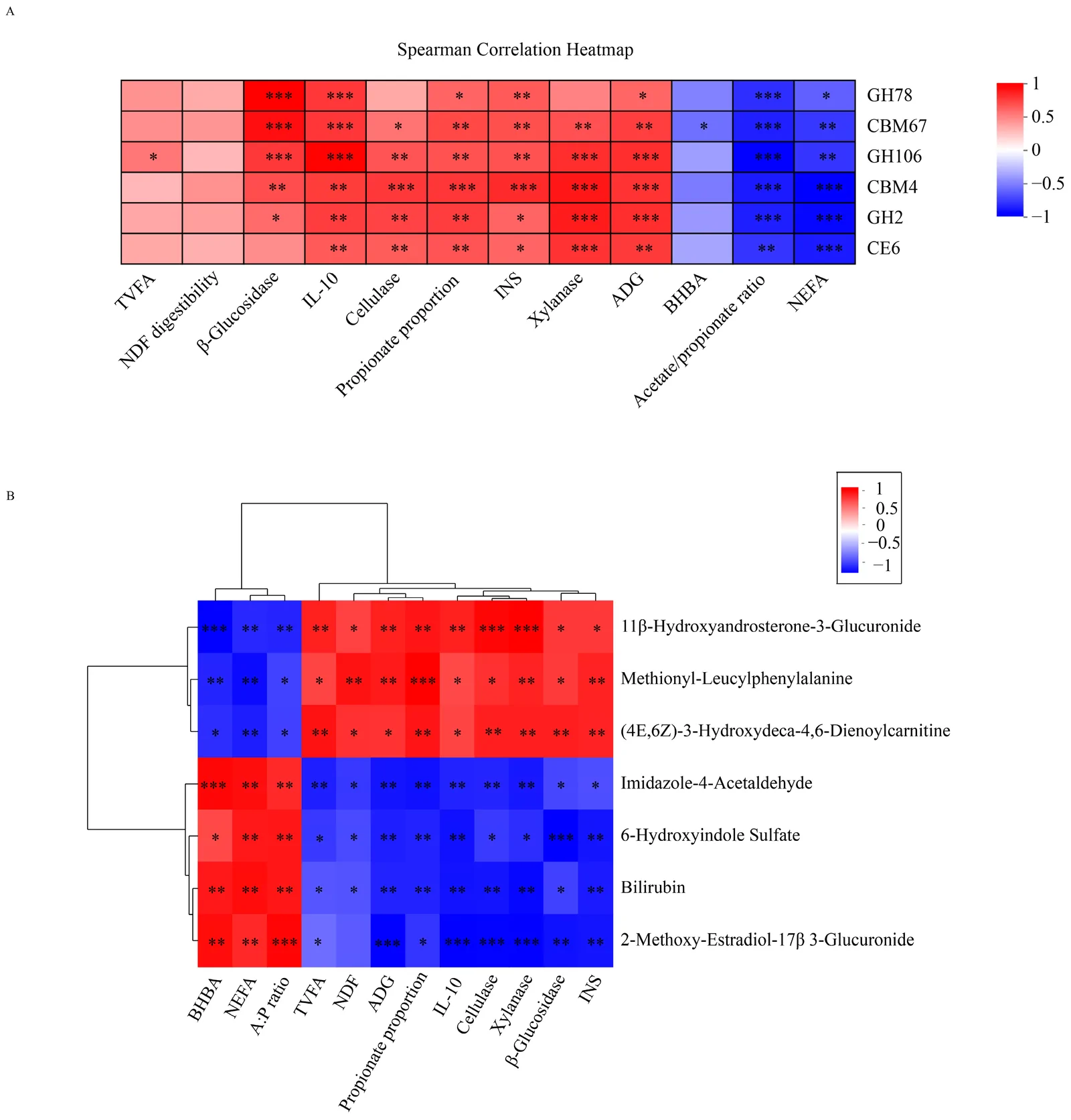
Correlations of representative CAZy families and key differential plasma metabolites with phenotypic traits. (**A**) Spearman correlation heatmap between representative CAZy families and digestive enzyme activities, rumen fermentation parameters, blood indices, nutrient digestibility, and growth traits. (**B**) Spearman correlation heatmap between key differential plasma metabolites and phenotypic traits. Colors represent Spearman correlation coefficients, with red indicating positive correlations and blue indicating negative correlations. Asterisks indicate correlations that remained significant after Benjamini–Hochberg false discovery rate correction (q < 0.05, * *p* < 0.05, ** *p* < 0.01, and *** *p* < 0.001). A:P ratio, acetate-to-propionate ratio; NEFA, non-esterified fatty acids; BHBA, β-hydroxybutyrate; INS, insulin; IL-10, interleukin-10; TVFA, total volatile fatty acid concentration; NDF digestibility, neutral detergent fiber digestibility.

**Figure 6:**
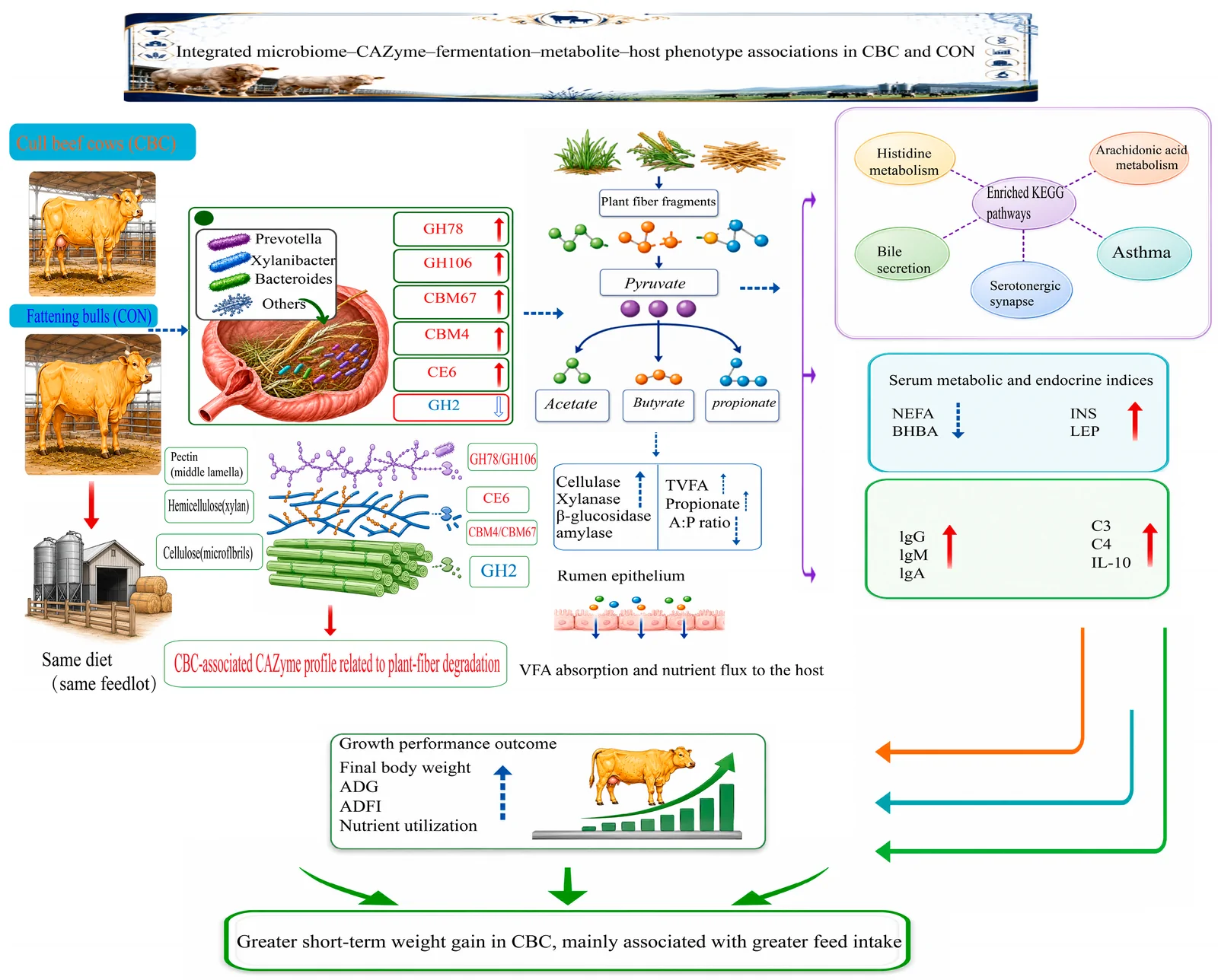
Descriptive microbiome–CAZyme–fermentation–metabolite–host phenotype framework summarizing associations observed between CBC and CON during fattening. The framework summarizes the observed associations among rumen microbial structure, CAZy family profiles, carbohydrate-degrading enzyme activities, rumen fermentation, plasma metabolic responses, blood biochemical and immune indices, and growth performance. Upward and downward arrows within the boxes indicate higher and lower values, respectively, in CBC relative to CON. Both solid and dashed connecting lines and arrows represent observed associations or schematic relationships rather than demonstrated causal relationships. CBC, culled beef cows; CON, fattening bulls.

**Table 1:** Ingredient composition and nutrient levels of the experimental diets (air-dry basis, %).

Items	%	Nutrient Composition (DM Basis)	%
Corn silage	23.5	Net energy (MJ/kg DM)	6.69
Corn stover	29.5	Crude protein (%)	11.1
Crushed corn	24.5	NDF (%)	38.8
DDGS	12.0	ADF (%)	22.1
Soybean meal	3.00	Calcium (%)	0.54
Cottonseed meal	3.00	Total phosphorus (%)	0.26
Urea	0.50		
Limestone	1.00		
Salt	0.80		
Sodium bicarbonate	0.60		
Dicalcium phosphate	0.60		
1% Beef cattle premix	1.00		
Total	100.00		

(1) DDGS = distillers dried grains with solubles. (2) The 1% beef cattle premix provided the following per kilogram: vitamin A 500,000 IU, vitamin D_3_ 150,000 IU, vitamin E 2400 IU, Fe 500 mg, Cu 1000 mg, Zn 2400 mg, Mn 1500 mg, Se 45 mg, and Co 15 mg. (3) DM = dry matter, CP = crude protein, NDF = neutral detergent fiber, ADF = acid detergent fiber, Ca = calcium, P = phosphorus. (4) Net energy was calculated based on the ingredients of the diet and their net energy content, rather than on the actual dry matter intake. (5) Ingredient composition is expressed on an air-dry basis, whereas nutrient levels are expressed on a dry matter basis. (6) Net energy was calculated from the ingredient composition and tabulated ingredient energy values according to the Chinese Beef Cattle Feeding Standard (NY/T 815-2004) [21], whereas CP, NDF, ADF, Ca, and P were determined by laboratory analysis. (7) The concentrate-to-forage ratio was approximately 45:55 on an air-dry basis after excluding urea, limestone powder, salt, sodium bicarbonate, dicalcium phosphate, and premix from the calculation.

**Table 2:** Growth performance and apparent nutrient digestibility in CON and CBC.

Variable	CON Mean ± SE	CBC Mean ± SE	*p*-Value
Growth performance			
Initial body weight (kg)	416 ± 2.35	417 ± 1.90	0.824
Final body weight (kg)	466 ± 2.29	476 ± 1.46	0.003
Average daily gain (kg/d)	0.83 ± 0.01	0.98 ± 0.02	<0.001
Average daily feed intake (kg/d)	9.12 ± 0.03	10.9 ± 0.10	<0.001
Feed-to-gain ratio	10.9 ± 0.11	11.1 ± 0.22	0.692
Apparent digestibility (%)			
Dry matter	69.4 ± 0.69	71.9 ± 0.56	0.012
Crude protein	65.1 ± 0.79	67.6 ± 0.75	0.040
Neutral detergent fiber	48.4 ± 0.79	51.3 ± 0.84	0.025
Acid detergent fiber	40.5 ± 0.37	41.6 ± 0.37	0.060

Values are presented as mean ± SE. CON, fattening bulls; CBC, culled beef cows.

**Table 3:** Within-group changes in body condition score of fattening bulls (CON) and culled beef cows (CBC).

Variable	CON Mean ± SE	CBC Mean ± SE
Initial body condition score (day 0)	5.44 ± 0.19	3.56 ± 0.18
Final body condition score (day 60)	7.06 ± 0.21	7.22 ± 0.15
Change in body condition score	1.61 ± 0.18	3.67 ± 0.20
Within-group *p*-value (day 0 vs. day 60)	0.004	0.004

Note: Values are presented as mean ± SE (*n* = 9 per group). Body condition score was assessed using a 1–9 scale. Within-group changes from day 0 to day 60 were evaluated using a two-sided Wilcoxon signed-rank test. Because different, non-cross-calibrated reference frameworks were used for CON and CBC, no between-group statistical comparisons of absolute BCS values or BCS changes were performed. Change in body condition score = final score − initial score.

**Table 4:** Economic evaluation of culled beef cows and fattening bulls during the feeding period.

Item	CON	CBC
Initial body weight (kg)	416	417
Final body weight (kg)	466	476
Live-weight gain over 60 d (kg/head)	50	59
Average daily feed intake (kg/day)	9.12	10.9
Total feed cost over 60 d (RMB/head)	793.44	944.82
Initial live-cattle price (RMB/kg)	28.00	24.00
Final live-cattle price (RMB/kg)	26.00	23.50
Change in live-animal market value under observed prices (RMB/head)	467.36	1180.30
Partial budget under observed prices ^1^ (RMB/head)	−326.08	235.48
Value of added live weight at a constant within-group price ^2^ (RMB/head)	1400.00	1416.00
Partial budget at a constant within-group price ^3^ (RMB/head)	606.56	471.18
Daily gain margin (RMB/head/day)	8.36	7.26
gain margin (RMB/head)	501.60	435.60

Note: CON, fattening bulls; CBC, culled beef cows; ADG, average daily gain; ADFI, average daily feed intake; TMR, total mixed ration. Economic calculations were based on market prices during November 2025 to January 2026. The TMR price was 1.45 RMB/kg DM. The initial live cattle prices were 28.0 RMB/kg for fattening bulls and 24.0 RMB/kg for culled beef cows, and the final live cattle selling prices were 26.0 RMB/kg for fattening bulls and 23.5 RMB/kg for culled beef cows. Daily feed cost = ADFI × TMR price. Daily gain revenue = ADG × final live cattle selling price. Daily gain margin = daily gain revenue − daily feed cost. Initial live cattle value = initial body weight × initial live cattle price. Final live cattle value = final body weight × final live cattle selling price. Body-value increment = final live cattle value − initial live cattle value. ^1^ Partial budget under observed prices = final body weight × final live cattle price − initial body weight × initial live cattle price − total feed cost over 60 d. ^2^ Value of added live weight at a constant within-group price = live-weight gain × initial live cattle price. ^3^ Constant-price partial budget = value of added live weight − total feed cost.

**Table 5:** Blood biochemical, hormonal, and immunological indices in CON and CBC.

Variable	CON Mean ± SE	CBC Mean ± SE	Raw *p*-Value	FDR-Adjusted q-Value
Serum biochemical indices				
GLU (mmol/L)	4.95 ± 0.14	4.91 ± 0.24	0.893	0.893
CREA (μmol/L)	76.3 ± 3.9	82.6 ± 3.2	0.232	0.313
NEFA (mmol/L)	0.322 ± 0.02	0.144 ± 0.01	<0.001	<0.003
BHBA (mmol/L)	0.58 ± 0.01	0.48 ± 0.03	0.012	0.023
TP (g/L)	73.3 ± 1.6	75.4 ± 2.4	0.474	0.582
ALB (g/L)	38.8 ± 0.6	39.2 ± 1.7	0.811	0.842
AST (U/L)	105 ± 6.19	107 ± 5.34	0.757	0.818
ALT (U/L)	30.7 ± 0.98	34.2 ± 2.07	0.156	0.263
UREA (mmol/L)	3.28 ± 0.09	3.68 ± 0.06	0.002	0.004
TC (mmol/L)	3.61 ± 0.11	4.71 ± 0.17	<0.001	<0.003
TG (mmol/L)	0.35 ± 0.02	0.38 ± 0.02	0.231	0.313
Serum hormone indices				
GH (ng/mL)	14.8 ± 0.34	12.4 ± 0.47	0.001	0.002
IGF-1 (ng/mL)	255 ± 6.89	238 ± 9.91	0.189	0.300
INS (ng/mL)	3.25 ± 0.23	4.48 ± 0.20	0.001	0.002
LEP (ng/mL)	10.4 ± 0.75	15.8 ± 0.71	<0.001	<0.003
E2 (pg/mL)	25.5 ± 1.56	30.6 ± 1.02	0.014	0.025
T (ng/mL)	3.97 ± 0.17	0.42 ± 0.37	<0.001	<0.003
Serum immunological indices				
IgG (mg/mL)	9.48 ± 0.60	12.9 ± 0.56	<0.001	<0.003
IgM (μg/mL)	920 ± 37.1	1660 ± 46.3	<0.001	<0.003
IgA (μg/mL)	672 ± 28.1	1260 ± 35.3	<0.001	<0.003
C3 (μg/mL)	67.4 ± 4.22	99.2 ± 3.77	<0.001	<0.003
C4 (μg/mL)	15.6 ± 0.83	20.2 ± 0.76	<0.001	<0.003
Hp (ng/mL)	1048 ± 23.7	1077 ± 21.4	0.387	0.498
SAA (μg/mL)	2.24 ± 0.15	2.38 ± 0.15	0.540	0.634
IL-6 (pg/mL)	40.9 ± 2.61	41.9 ± 0.79	0.724	0.815
IL-10 (pg/mL)	58.8 ± 2.47	74.5 ± 1.92	<0.001	<0.003
TNF-α (pg/mL)	166.1 ± 1.27	170.8 ± 3.27	0.207	0.311

Note: Values are presented as mean ± SE. Raw *p*-values are from the between-group comparisons. The 27 blood biochemical, hormonal, and immunological indices were treated as a single family and adjusted using the Benjamini–Hochberg false discovery rate procedure. Statistical significance after correction was defined as q < 0.05. For variables with raw *p* < 0.001, the corresponding FDR-adjusted q-values were <0.003. CON, fattening bulls; CBC, culled beef cows; GLU, glucose; CREA, creatinine; NEFA, non-esterified fatty acids; BHBA, β-hydroxybutyrate; TP, total protein; ALB, albumin; AST, aspartate aminotransferase; ALT, alanine aminotransferase; UREA, urea; TC, total cholesterol; TG, triglyceride; GH, growth hormone; IGF-1, insulin-like growth factor 1; INS, insulin; LEP, leptin; E2, estradiol; T, testosterone; IgG, immunoglobulin G; IgM, immunoglobulin M; IgA, immunoglobulin A; C3, complement C3; C4, complement C4; Hp, haptoglobin; SAA, serum amyloid A; IL-6, interleukin-6; IL-10, interleukin-10; TNF-α, tumor necrosis factor-α.

**Table 6:** Summary statistics of representative CAZy families between CON and CBC.

Family	CON Mean ± SE (%)	CBC Mean ± SE (%)	*p*-Value
GH2	70.3 ± 0.96	66.6 ± 0.61	0.004
GH106	10.7 ± 0.28	11.2 ± 0.09	0.060
GH78	9.34 ± 0.63	11.1 ± 0.53	0.012
CBM67	3.40 ± 0.24	4.06 ± 0.12	0.016
CBM4	3.17 ± 0.05	3.66 ± 0.06	<0.001
CE6	2.99 ± 0.12	3.42 ± 0.14	0.005

Values are presented as mean ± SE. CON, fattening bulls; CBC, culled beef cows; CAZy, Carbohydrate-Active enZYmes database; GH, glycoside hydrolases; CBM, carbohydrate-binding modules; CE, carbohydrate esterases. Direction/level indicates the group with higher relative abundance and the statistical level of the difference.

**Table 7:** Top contributing genera for representative CAZy families.

Family	Top Contributor 1	Top Contributor 2	Top Contributor 3
GH2	*Prevotella* (1795)	Others (981)	*Xylanibacter* (558)
GH106	*Prevotella* (247)	Others (223)	*Bacteroides* (63)
GH78	Others (287)	*Prevotella* (134)	*Bacteroides* (65)
CBM67	Others (129)	*Prevotella* (43)	*Bacteroides* (24)
CBM4	*Prevotella* (109)	Others (42)	*Xylanibacter* (14)
CE6	*Prevotella* (77)	Others (63)	*Xylanibacter* (52)

Contributors were ranked within each representative CAZy family according to platform-derived species-function contribution values. The top three contributors are shown with contribution values in parentheses. Genus names are italicized. “Others” represents taxa grouped by the analysis platform rather than a single genus.

**Table 8:** Key differential plasma metabolites matched to enriched pathway categories between CON and CBC.

Metabolite	Regulation in CBC	FC	VIP	Raw *p*-Value	FDR-Adjusted q-Value	Annotation Level	Matched Pathway/Biochemical Category	Main Implication
11B,13-Dihydrolactucin	Down	0.639	4.59	0.00129	0.0216	B(ii)	Terpene lactone-related metabolism	Terpene-lactone-related
Methionyl-Leucylphenylalanine	Up	1.591	4.40	8.89 × 10^−4^	0.01663	B(i)	Amino acid/peptide metabolism	Peptide-related
Bilirubin	Down	0.763	4.28	3.26 × 10^−5^	0.00178	B(i)	Bile pigment/hepatobiliary metabolism	Bile-pigment-related
N-Lauroyl Cysteine	Up	1.459	4.18	0.00269	0.03588	B(ii)	Amino acid/lipid-conjugate-related metabolism	Amino-acid/peptide-related
Mono-(2-Ethyl-5-Oxohexyl) Phthalate	Down	0.719	4.04	7.46 × 10^−6^	7.57 × 10^−4^	B(ii)	Phthalate-related metabolism	Phthalate-related
6-Hydroxyindole Sulfate	Down	0.769	3.75	3.80 × 10^−6^	5.23 × 10^−4^	B(ii)	Tryptophan/indole-related metabolism	Indole/sulfate-related
2,4-Dihydroxy-3,5,6-Trimethylbenzoic Acid	Down	0.805	3.66	1.33 × 10^−6^	2.71 × 10^−4^	B(i)	Benzoic acid-related metabolism	Benzoic-acid-related
(4E,6Z)-3-Hydroxydeca-4,6-Dienoylcarnitine	Up	1.323	3.65	0.00219	0.03107	B(ii)	Fatty acid oxidation/acylcarnitine-related lipid metabolism	Acylcarnitine-related lipid metabolite
2-Methoxy-Estradiol-17β 3-Glucuronide	Down	0.819	3.52	2.65 × 10^−6^	4.33 × 10^−4^	B(ii)	Steroid hormone biosynthesis	Steroid/glucuronide metabolism
4-Bis(2-Hydroxyethyl)Amino-L-Phenylalanine	Up	1.262	3.44	4.11 × 10^−4^	0.01003	B(ii)	Amino acid-related metabolism	Amino-acid-related
(−)-Gossypol	Down	0.798	3.40	0.00339	0.04194	B(i)	Gossypol-related metabolism	Gossypol-related
Imidazole-4-Acetaldehyde	Down	0.761	3.40	0.00279	0.03695	B(ii)	Histidine metabolism	Histidine metabolism
11β-Hydroxyandrosterone-3-Glucuronide	Up	1.201	3.29	1.84 × 10^−5^	0.00126	B(ii)	Biosynthesis of cofactors; entose and glucuronate interconversions; ile secretion; scorbate and aldarate metabolism; ryptophan metabolism	Glucuronide/hepatobiliary metabolism
Paracetamol Sulfate	Up	1.220	3.25	1.06 × 10^−5^	9.25 × 10^−4^	B(i)	Sulfate-conjugation-related metabolism	Sulfate-conjugated metabolite

Note: FC, fold change; VIP, variable importance in projection. Values were obtained from the animal-level reanalysis using six biological replicates per group after technical injections were combined for each animal. “Up” and “Down” indicate higher and lower relative abundance, respectively, in CBC than in CON. Key differential metabolites were defined as q < 0.05, VIP > 1, and FC > 1.20 or FC < 0.83. Numerical values are reported to approximately three significant figures. Some pathway groups represent biochemical-class interpretations rather than direct KEGG pathway annotations. Annotation confidence was reported using the Majorbio platform-specific scheme: B(i), matching against experimental MS/MS spectral libraries (in-house or commercial); B(ii), matching against computer-simulated theoretical MS/MS spectral libraries. These annotations were based on spectral-library matching and were not confirmed using authentic reference standards.

## Data Availability

The rumen metagenomic sequencing data generated in this study have been deposited in the NCBI Sequence Read Archive (SRA) under BioProject accession number PRJNA1481135. The processed plasma metabolomics feature table and raw LC–MS data are currently being deposited in a public metabolomics repository. The repository name and accession number will be added upon completion of the deposition process.

## Supplementary Materials

The following supporting information can be downloaded at https://www.mdpi.com/article/10.3390/ani16193052/s1. Table S1. Sensitivity of the partial budget to alternative live-cattle price scenarios.
