# Supplementary material for: Comparative Study on Growth Performance and Rumen Digestive Metabolism Between Cull Beef Cows and Fattening Bulls
Source: Animals (Basel). 2026 Sep 28;16(19):3052. doi: 10.3390/ani16193052 (PMC13663404; doi:10.3390/ani16193052)
Supplement: Supplementary file 1 [file animals-16-03052-s001.zip › animals-4468398-supplementary.pdf]

Table S1. Sensitivity of the partial budget to alternative live-cattle price scenarios

**Price scenario CON (RMB/head)** Observed		**CBC (RMB/head)**	**Higher value**
price path −326.08		235.48	CBC
No price change in either group	606.56	471.18	CON
5% price decline in both groups	-45.84	-100.02	CON
7.14% price decline in both groups	-325.44	-344.82	CON
10% price decline in both groups	-698.24	-671.22	CBC

Note: Animal performance and feed cost were held constant in the sensitivity analysis, while live-cattle price trajectories were varied. The change in relative ranking across scenarios indicates that the economic comparison is sensitive to market-price assumptions and should not be attributed solely to animal performance.
